# Imbalance in chemical space: How to facilitate the identification of protein-protein interaction inhibitors

**DOI:** 10.1038/srep23815

**Published:** 2016-04-01

**Authors:** Mélaine A. Kuenemann, Céline M. Labbé, Adrien H. Cerdan, Olivier Sperandio

**Affiliations:** 1 Université Paris Diderot, Sorbonne Paris Cité, Molécules Thérapeutiques In Silico, INSERM UMR-S 973, Paris, France; 2 INSERM, U973, Paris, France

## Abstract

Protein-protein interactions (PPIs) play vital roles in life and provide new opportunities for therapeutic interventions. In this large data analysis, 3,300 inhibitors of PPIs (iPPIs) were compared to 17 reference datasets of collectively ~566,000 compounds (including natural compounds, existing drugs, active compounds on conventional targets, etc.) using a chemoinformatics approach. Using this procedure, we showed that comparable classes of PPI targets can be formed using either the similarity of their ligands or the shared properties of their binding cavities, constituting a proof-of-concept that not only can binding pockets be used to group PPI targets, but that these pockets certainly condition the properties of their corresponding ligands. These results demonstrate that matching regions in both chemical space and target space can be found. Such identified classes of targets could lead to the design of PPI-class-specific chemical libraries and therefore facilitate the development of iPPIs to the stage of drug candidates.

Protein-protein interactions (PPIs) play essential roles in nearly all biological processes, and their deregulation is often associated with disease states. Therefore, there is a growing interest to target PPIs for therapeutic interventions not only with biologics but also using low-molecular-weight (LMW) compounds (<1,000 g.mol^−1^). Still, targeting PPIs with LMW drugs remains one of the most difficult challenges in molecular medicine^1^. Although great innovations have been achieved to facilitate the identification of inhibitors of PPI targets (iPPIs) (e.g., fragment-based drug design, Nuclear Magnetic Resonance (NMR), X-Ray crystallography, etc.), experimental screening procedures for PPI targets still suffer from the unavailability of suitable fragments and chemical libraries^2^,^3^,^4^. Indeed, the molecular topography of most known PPIs, which are often described as shallow, large, and hydrophobic, makes them harder to tackle with small compounds. This situation has often been translated into designing larger, more hydrophobic and more aromatic compounds^2^,^3^,^4^. Such compounds dramatically diminish the likelihood of obtaining a safe and specific drug at the end of the development process^5^,^6^,^7^. In addition to such impeding properties, other studies have nevertheless highlighted specific physicochemical characteristics that may be necessary for iPPIs to bind PPI interfaces. These characteristics include the specific 3D shapes^8^,^9^,^10^ of those compounds. Recently, our group has identified new 3D characteristics of inhibitors of PPI targets^11^. In this seminal analysis, four shape properties were shown to be specific to the structure of iPPI compounds, including the globularity (glob) and the Volsurf^12^ properties EDmin3, CW2, and IW4 in a distribution of putative hydrophobic and hydrophilic interacting regions around the compound. Most noticeably, EDmin3, which describes the capacity of a compound to efficiently bind the hydrophobic patch that is often present at the core of a PPI interface, is an important structural feature for nearly all iPPI compounds regardless of the heterogeneity of the PPI target space. In contrast to the previously identified shape features^8^,^9^, such properties correlate with neither the size nor the hydrophobicity of the compounds and could therefore allow chemists to prioritize the selection of PPI-compliant LMW compounds without having to drive potency through molecular obesity^5^.

The goal of this study was to capitalize on this cumulative knowledge to obtain further insight into the iPPI chemical space and to evaluate the heterogeneity of the known PPI target space. We aimed to establish a proof-of-concept that some classes of known PPI targets can be identified either by detecting shared properties and chemotypes for the ligands meant to modulate them or by analysing the properties of the PPI interfaces’ binding cavities. This identification would facilitate the design of PPI-class-specific chemical libraries and would boost the identification of active compounds on PPI targets.

To this end, we have combined iPPI compounds and pharmacological data from both iPPI-DB^13^ and TIMBAL^14^. To the best of our knowledge, this study is the first of its kind, applying such a wide dataset of iPPI compounds (~3,250 representative compounds after preparation) across 29 PPI targets. We also selected a series of annotated libraries as reference chemical datasets that contain compounds that are not considered iPPIs (called hereafter non-iPPIs): natural compounds, allosteric compounds, advanced drug candidates, launched drugs, active compounds on conventional targets, and compounds from commercial libraries. This series collectively corresponds to 566,000 non-iPPI compounds. Furthermore, we selected particular group molecular descriptors known to be iPPI-selective, namely the octanol/water partition coefficient (AlogP), the molecular weight, the aromatic ratio (proportion of aromatic atoms) and the 4 shape descriptors cited above (glob, EDmin3, CW2, and IW4). These descriptors were used to define for these joined datasets the foundations of a chemical space in which iPPIs can be more suitably distinguished from non-iPPIs. These iPPI-selective descriptors were combined with conventional descriptors that are commonly used to depict chemical space. The resulting chemical space was then visualized and analysed using a principal component analysis (PCA). Further along this line, a new metric is described hereafter to quantify the overlap in chemical space between two populations of compounds. This metric uses the probability density functions for each dataset along with each principal component of a PCA calculated from joined sets of compound libraries and a given set of descriptors. The use of such an approach has confirmed several known trends about the iPPI chemical space compared to conventional molecules, such as their overall higher size, higher aromaticity, and higher hydrophobicity. This approach supports the selective character of the 4 shape descriptors described above (glob, EDmin3, CW2, and IW4). However, this approach also underpins novel observations, such as the important heterogeneity of the PPI target space. Indeed, this study allowed us to create some classes of PPI targets by comparing the properties of their corresponding ligands. The determination of such ligand-driven PPI classes was then compared to the classes that were obtained using a purely pocket-driven analysis of the PPI targets themselves. Most of the PPI target classes could thus be confirmed using this latter orthogonal approach. Therefore, as a proof of concept, we show trends that confirm some of the observations that were made using the iPPI compounds alone and provide a perspective with which to generalize such pocket-driven approaches PDB-wide^15^ (Protein Data Bank). Indeed, it seems that not only can the properties of binding pockets be used to group PPI targets, but they can certainly condition the properties of their corresponding ligands in a predictable manner.

## Results and Discussion

### Description of the datasets

For the present analysis, we utilized a set of iPPI compounds and a set of non-iPPI compounds. For iPPIs, we combined the compounds and binding data of the iPPI-DB and TIMBAL databases that contain, after standardization, a total of 3,248 non-redundant iPPIs across 29 PPI targets: 1,650 from iPPI-DB across 13 targets, and 1,598 iPPI from TIMBAL across 16 other targets (Fig. S1). We chose to apply the standards of iPPI-DB to the compounds of TIMBAL in order to maximize the homogeneity of the data, including the availability of binding data in the form of a XC_50_ (e.g., IC_50_, K_d_, EC_50_, K_i_) inferior to 30 μM, the existence of a clearly identified PPI target (e.g., excluding TIMBAL’s Integrins), and the exclusion of all peptides (defined as 3 sequential peptide bonds) and of macrocycles. The present procedure was used to limit the presence of non-specific binders and to focus on small molecules (molecular weight <1,200 g.mol^−1^).

For the sets of non-iPPI compounds, we selected different databases. The goal was to better represent the diversity of compounds that can be considered negative data with respect to iPPIs. The list of those datasets is presented in Table 1 and refers to different types of small molecules: active compounds under development, actual drugs, allosteric modulators, natural compounds, or compounds from commercial chemical libraries. From MDDR (www.biovia.com), we gathered compounds that are presently under development, either in biological testing (early development), preclinical phases, and clinical phases or launched drugs. Along the same lines, we collected a subset of drugs that are orally bioavailable from the E_Drugs3D database^16^, called e_Drug hereafter. We also collected the allosteric modulators of the ASD database^17^ or a selection of conventional inhibitors that are active in the top 100 most studied targets from BindingDB^18^ (version 2012), which are all conventional targets, i.e., GPCRs (G-protein coupled receptors), enzymes, kinases, ion channels, or nuclear receptors. We also selected a chemically diverse set of compounds, BDM, from three different commercial chemical libraries: Asinex^19^ (v2012), ChemDiv^20^ (v2012) and Enamine^21^ (v2012). Finally, we selected the natural compounds of the Nubbe database^22^. Together, this overall set of 566,208 non-iPPI compounds represents a negative dataset for iPPI compounds and provides a comparative analysis of the physicochemical properties of iPPIs with respect to the different types of annotated compounds. This dataset will facilitate us obtaining a global positioning system in chemical space for iPPI compounds. To treat equally positive (iPPIs) and negative (non-iPPI compounds) data, all of the datasets of iPPI compounds and non-iPPI compounds were standardized using the exact same procedure (see the standardization procedure in Methods).

### Assessment of conventional ADME/tox rules and iPPI prediction rules

We first estimated the drug-like profile of each dataset using in silico routines. To this end, we ran the FAF-Drugs3 web server^23^ to measure and compare different physicochemical rules that are commonly used to predict the pharmacokinetic profiles of drug candidates. These rules include PAINS-containing substructures^24^; rules for drug likeness and/or bioavailability, such as Lipinski’s RO5^25^, Veber’s^26^, Eli Lilly^27^, Gleeson’s X4_400^28^, and Egan’s^29^; and some predictive models for iPPI compounds, such as PPI-HitProfiler^9^ and the 2P2I RO4^30^.

The detection of PAINS substructures (Fig. S2) was performed for all of the compounds from all of the datasets by differentiating the three established filters: PAINS-A, PAINS-B and PAINS-C. Those filters were created by Baell *et al*.^24^ to consider three levels of pan-assay-interfering substructures depending on the number of occurrences, such chemical moieties, that were observed in their experimental screenings over the years. More precisely, the most problematic substructures are listed in PAINS-A: they have been observed to be active in more than 150 screening campaigns. The substructures from PAINS-B have been observed in experimental assays between 15 and 150 times. Finally, the substructures from PAINS-C have been observed fewer than 15 times. The present PAINS analysis indicates that iPPIs tend to show on average more PAINS-containing compounds (20% for iPPI-DB, mostly from filter PAINS-C, and 15% for TIMBAL, mostly from filter PAINS-A) than active compounds on conventional targets (less than 10%, except for kinase with 15%). Nevertheless, this pattern is not the case for all of the PPI targets. Indeed, Bcl-2, MDM2, CD80, ITGAL and E1 are the PPI targets that have the highest proportions of PAINS-containing compounds, although not necessarily for the same filter (PAINS-A, PAINS-B, or PAINS-C). Interestingly among the detected PAINS substructures, for example, among the Bcl-2 inhibitors, one can find catechol from filter PAINS-B. However, this chemical moiety is also found in gossypol or apogossypol, which are currently in clinical trials as treatments for leukaemia. Thus, for conventional inhibitors, most of the iPPI compounds are not predicted as promiscuous binders or frequent hitters according to Baell’s PAINS.

The level of compliance of all of the datasets with respect to all of the remaining chemistry rules cited above confirms that iPPIs exhibit different profiles compared to conventional drug-like compounds (Fig. S3). The use of such rules on orally bioavailable drugs, such as e_Drug compounds, highlights the importance of the Lipinski’s RO5, Veber’s, Gleeson’s X4_400, Egan’s and Eli Lilly’s compounds to estimate the drug-likeness or the bioavailability of compounds. In contrast, both iPPI-DB and TIMBAL compounds have the worst profiles according these rules, underpinning the now known hydrophobic character and the larger size of iPPIs with respect to active compounds on conventional targets (GPCR, enzyme, etc.) or drug candidates, as most of these rules partially rely on molecular weight and logP (octanol/water partition coefficient). However, one concern is the compliance of all of these rules in the iPPI datasets when subdividing them according to the activity of the compounds (Fig. S4). Indeed, when considering two bins of activity, namely pXC50 < 7 or pXC50 > 7, the chemistry rules penalize the most potent iPPI compounds even more dramatically. Again, most of these rules partially rely on molecular weight and hydrophobicity (i.e., octanol/water partition coefficient, or logP), indicating that there is still a price to pay for iPPI in terms of size and hydrophobicity to reach a critical level of potency, impeding the development of drug candidates.

Finally, we also assessed known predictive models of iPPI, such as PPI-HitProfiler^9^ and 2P2I RO4^30^, on the iPPI dataset. Those models confirm their good levels of predictability (from 70 to 92%) on the iPPI datasets and their satisfactory specificity with respect to non-iPPI datasets (Figs S3 and S4).

### Confirmation of known iPPI specific descriptors

**Figure 1:**
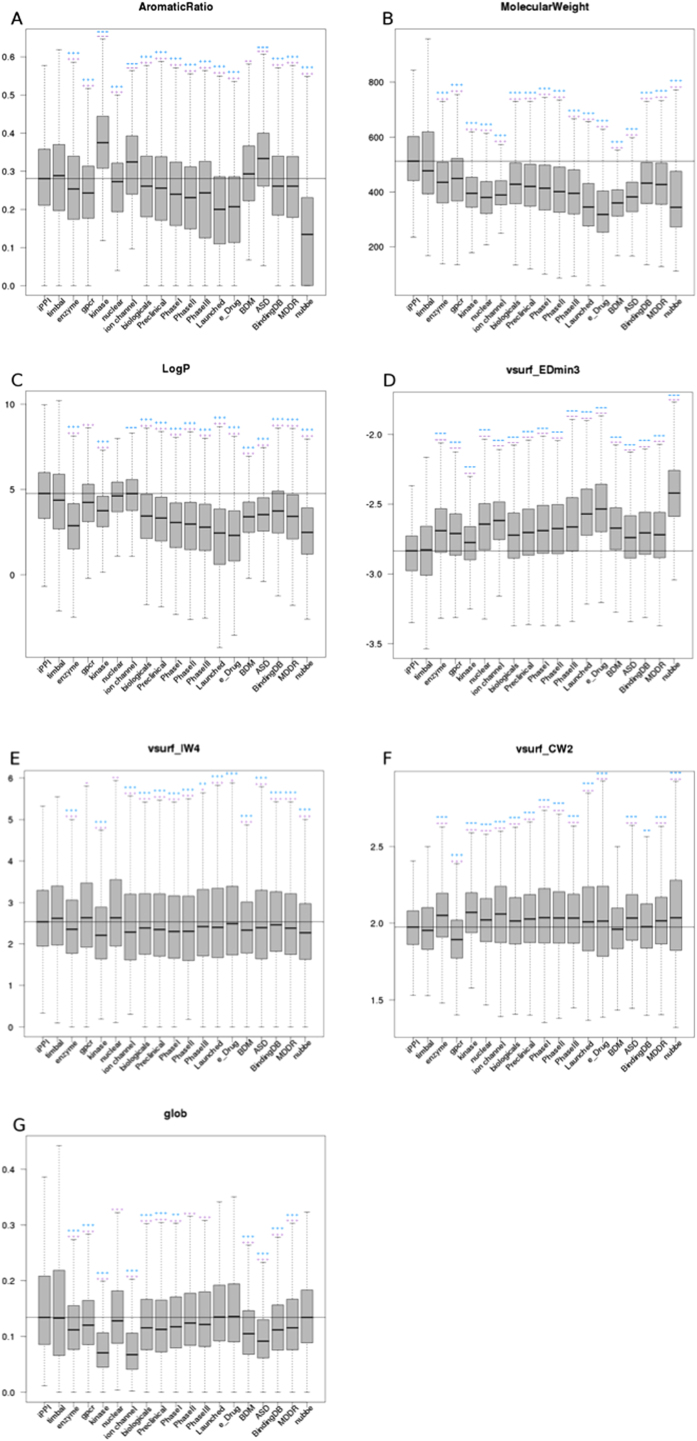
Distribution of all of the datasets of the seven properties: (**A**) aromatic ratio, (**B**) molecular weight, (**C**) ALogP, (**D**) EDmin3, (**E**) IW4, (**F**) CW2 and (**G**) globularity (glob). Stars above the bar plots represent the level of significance resulting from a pairwise comparison of the iPPI datasets (iPPI-DB and TIMBAL) versus all of the non-iPPI datasets, from *moderately significant (0.01 < P-value < 0.05), **significant (0.001 < P-value < 0.01), to ***very significant (P-value < 0.001). The colour code for the stars depicts the test that was carried out in reference to iPPI-DB (violet) or to TIMBAL (blue).

We then used the datasets of iPPI compounds (iPPI-DB + TIMBAL) and non-iPPI compounds (all remaining datasets) to evaluate the levels of specificity of already known discriminative descriptors towards iPPI and to further confirm the results of our recently reported work^11^. To this end, we tested the following descriptors: molecular weight (MW), hydrophobicity (AlogP), proportion of aromatic atoms (aromatic ratio), and the 4 shape descriptors cited above: globularity (glob), CW2, IW4, and EDmin3. To measure the levels of specificity of those descriptors with respect to non-iPPI compounds, we ran an ANOVA (Analysis Of Variance) followed by post hoc comparisons using a pairwise test (see Methods) between the iPPI datasets and all of the individual non-iPPI datasets (Fig. 1).

As a global trend, iPPI compounds display a significantly higher molecular weight (MW_iPPI-DB_ = 540 g mol^−1^ ± 145; MW_Timbal_ = 521 g mol^−1 ^± 179) than that of all of the non-iPPI datasets. Compared to most of the non-PPI datasets, iPPIs present a significantly higher hydrophobicity (AlogP_iPPI-DB_ = 4.61 ± 1.98; AlogP_Timbal_ = 4.19 ± 2.39), except for ion channel inhibitors (AlogP_ion channel_ = 4.64 ± 1.52). The aromatic ratio, i.e., the proportion of aromatic atoms in a molecule, is most often significantly higher for iPPI compounds (aromatic ratio_iPPI-DB_ = 0.29 ± 0.10; aromatic ratio_TIMBAL_ = 0.29 ± 0.12), except when compared to ion channel, BDM, ASD, and kinase compounds (aromatic ratio_ion channel_ = 0.32 ± 0.10; aromatic ratio_BDM_ = 0.29 ± 0.11; aromatic ratio_ASD_ = 0.33 ± 0.11; aromatic ratio_kinase_ = 0.38 ± 0.10), for which significantly higher values have been found. Furthermore, iPPIs tend to have significantly lower values of CW2 (CW2_iPPI-DB_ = 1.98 ± 0.17; CW2_Timbal_ = 1.97 ± 0.20) than do non-iPPI compounds, except for GPCR compounds (CW2_GPCR_ = 1.9 ± 0.18), which have even more significantly lower values. This property illustrates that the ratio between the hydrophilic regions is defined at −0.5 kcal.mol^−1^ and the molecular surface of the molecule. Thus, it seems that iPPIs have on average smaller proportions of exposed soft polar regions than the compounds of non-iPPI datasets. In contrast, iPPIs tend to have significantly higher values of IW4 (IW4_iPPI-DB_ = 2.69 ± 1.00; IW4_TIMBAL_ = 2.78 ± 1.16) than do non-iPPI compounds, except for GPCRs (IW4_GPCR_ = 2.78 ± 1.15) and nuclear receptor compounds (IW4_Nuclear_ = 2.87 ± 1.31), which have significantly higher values of IW4 than do iPPIs. This property illustrates the distance between the centre of mass of the molecule and the barycentre of its polar regions defined at −2 kcal.mol^−1^. This property therefore expresses a higher degree of concentration of those soft polar regions at one extremity of the molecule, most often in the case of iPPIs, with the exception of GPCRs and nuclear receptor compounds. Globularity has also been confirmed as an important factor for iPPI. These compounds are indeed on average significantly more globular (Glob_iPPI-DB_ = 0.16 ± 0.09; Glob_Timbal_ = 0.15 ± 0.11) than are all of the non-iPPI datasets, except for existing drugs, i.e., MDDR/launched (Glob_Launched_ = 0.15 ± 0.08), e_Drugs (Glob_eDrugs_ = 0.15 ± 0.09), and natural compounds (Glob_Nubbe_ = 0.15 ± 0.09). This result therefore confirms the importance of having chemical structures with a higher isotropic occupation of the 3D space. Interestingly, natural compounds, which are defined by a higher number of sp3 carbon atoms and a higher level of chirality and complexity, have a globularity that is not significantly distinguishable from those of iPPI compounds. Furthermore, existing drugs have on average a globularity that is in the same range as those of iPPI compounds. This pattern is not the case for drug candidates in Phases I, II and III. Indeed, these compounds have a significantly lower globularity than do iPPI compounds, confirming the importance of discerning suitable molecular shapes when designing drugs and the concept that particular molecular shape is definitely worth investigating as an alternative to driving potency through molecular obesity^5^. Finally and most importantly, the property EDmin3, which describes the capacity of a compound to efficiently bind a hydrophobic patch at a protein surface, has been confirmed for iPPI compounds as significantly lower in energy (i.e., more efficient) (EDmin3_iPPI-DB_ = −2.85 kcal.mol^−1^ ± 0.19; EDmin3_Timbal_ = −2.84 kcal.mol^−1^ ± 0.26) than all of the non-iPPI datasets. This property still appears to be key in the binding of such compounds to their respective PPI targets.

### Discrepancies among PPI targets

**Figure 2:**
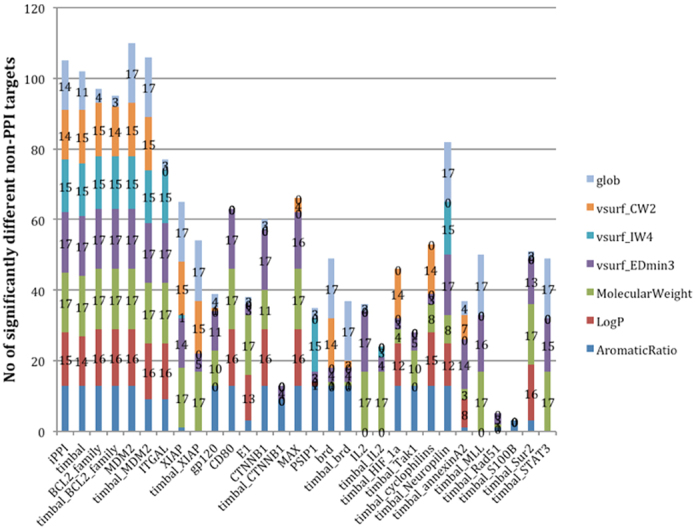
Stacked histogram showing for each PPI target the number of non-iPPI datasets for which a given descriptor is significantly different. For example, the iPPI compounds of Xiap have a significantly lower mean value of EDMin3 than that of 14 different non-iPPI datasets. The figure provides an overall evaluation of the discriminative power of the descriptors (height of each peak) and for which descriptor this pattern is the case (each different component of each peak).

To address the heterogeneity of the PPI target space first using iPPI properties and to ensure that those properties are not specific to the PPI targets that have the highest numbers of iPPI compounds, we also compared the distributions of those descriptors for each PPI target individually (28 PPI families from TIMBAL and iPPI-DB that have more than 5 iPPI compounds) by running an ANOVA followed by post hoc comparisons using a pairwise test (see Methods). Indeed, the distribution of each descriptor for each PPI target was compared to that of each non-iPPI dataset. Each significant difference with respect to a non-iPPI dataset was counted and plotted (Fig. 2). The most frequent discriminative descriptors were again the molecular weight and EDmin3, which are significantly different with respect to the compounds of non-iPPI datasets for most of the PPI targets. To a lesser extent, the globularity, aromatic ratio and AlogP were also significantly different from those of the non-iPPI datasets for a significant number of PPI targets. The other properties, CW2 and IW4, display good discriminative power but only for some PPI targets, namely Bcl2, MDM2, ITGAL, Xiap, PSIP1, bromodomain, HIF-1A, and neuropilin. Interestingly, CW2, IW4, and globularity may constitute surrogate descriptors for EDmin3 for the rare PPI targets; this property does not fall into the expected PPI profile. Indeed, it seems that alternatively for timbal_Xiap, PSIP1, HIF-1A and cyclophilin, these descriptors are discriminative with regards to most of the non-iPPI datasets, while EDmin3 is not. Finally for only 6 PPI targets out of 28 (E1, timbal_CTNNB1, timbal_IL2, timbal_tak1, timbal_Rad51, and timbal_S100B), none of the identified 3D descriptors were significantly different from a substantial number of non-iPPI datasets. Thus, for most of the known iPPI compounds, the above-cited 3D descriptors were specific by themselves, such as EDmin3; as a combination; or as surrogate descriptors for EDmin3, such as globularity, IW4, and CW2. In contrast, when plotting the discriminative power of the descriptors with reference to the non-iPPI datasets, EDmin3 was again confirmed as a significantly specific descriptor with respect to PPI targets (Fig. S5), confirming the importance of that property despite the heterogeneity of the PPI target space.

**Figure 3:**
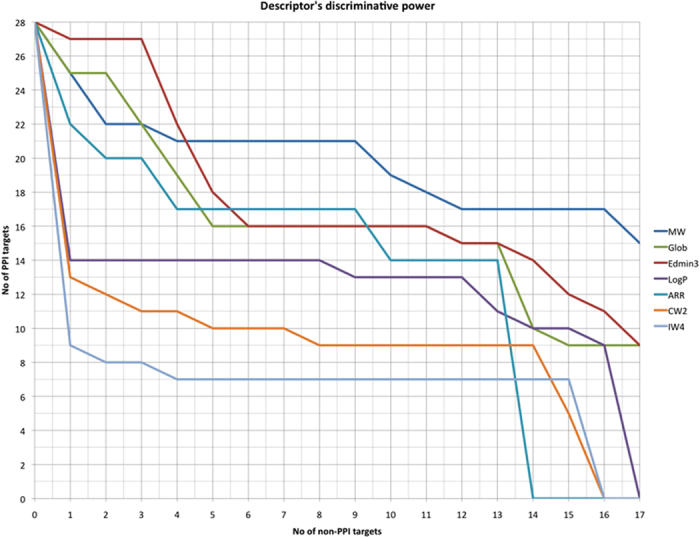
Number of PPI targets as a function of the number of significantly different non-iPPI datasets. For example, the iPPIs of at most 27 PPI targets had a significantly different EDmin3 (lower in the case of EDmin3) than did the compounds of 3 non-iPPI datasets, and the iPPI compounds of at most 21 PPI targets had a significantly different molecular weight (higher in the case of molecular weight) than did the compounds of 9 non-iPPI datasets.

To further confirm this trend, we plotted for each descriptor (globularity (glob) EDmin3, IW4, CW2, molecular weight (MW), AlogP, and aromatic ratio) the number of PPI targets as a function of the number of non-iPPI datasets for which they are significantly different (Fig. 3). For example, the compounds of 27 PPI targets had a significantly lower value of EDmin3 than that of 4 non-iPPI datasets, and the compounds of 21 PPI targets had a significantly higher molecular weight than that of 9 non-iPPI datasets. It is clear from Fig. 3 that the molecular weight of iPPI remained significantly higher for most of the PPI targets with respect to most of the non-iPPI datasets.

Nevertheless, globularity and, more importantly, EDmin3 are among the most significantly specific descriptors of iPPI compounds, even more so than the AlogP or aromatic ratio. Indeed, EDmin3 seems to better characterize the specificity of iPPI compounds. Finally, because CW2 and IW4 are significantly different only for a subset of PPI targets, their discriminative power as a global trend is not as important. These descriptors nevertheless remain extremely efficient when used to characterize the appropriate PPI targets and must therefore be kept and used to help to address the PPI target space heterogeneity. These 3D properties therefore provide us with a set of descriptors to separate iPPIs from non-iPPI compounds as efficiently as the hydrophobicity, aromaticity and molecular weight of the compounds.

### Visualization of the iPPI chemical space

**Figure 4:**
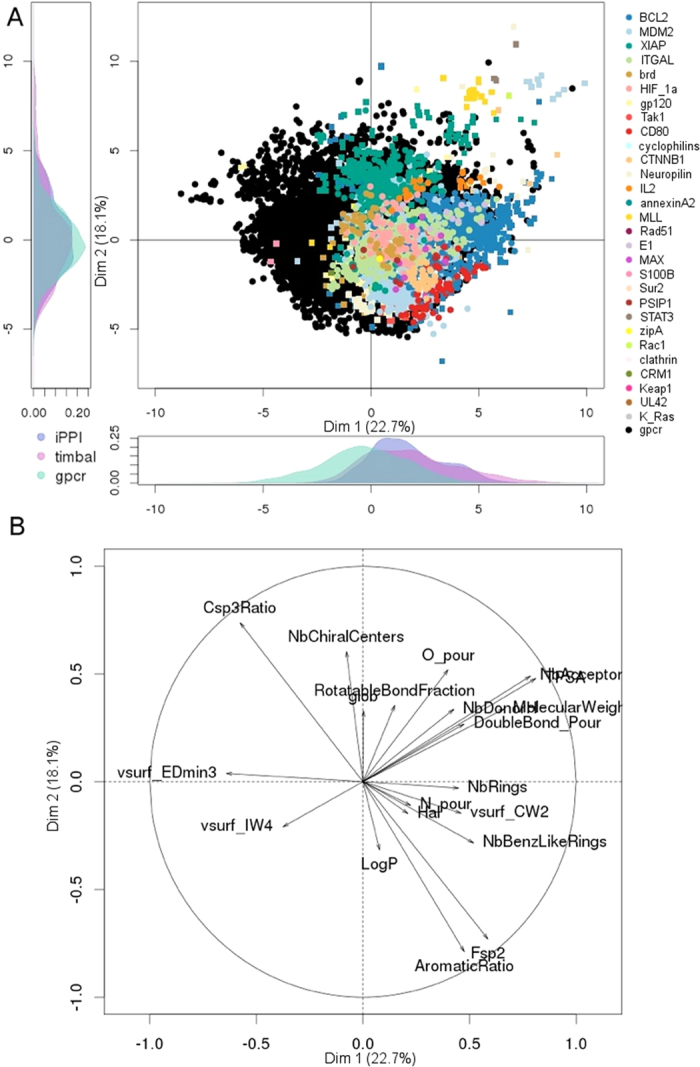
Principal component analysis calculated for all of the datasets. For clarity’s sake, only the iPPI datasets (iPPI-DB as coloured circles and TIMBAL as coloured squares) and the GPCR dataset (as black circles) from BindingDB are shown. (**A**) Individual map of compounds. (**B**) Variable map, circle of correlation.

Having identified discriminative descriptors to properly distinguish iPPIs from non-iPPI compounds, we then attempted to visualize the joined chemical space comprising all of the datasets. A common and convenient tool with which to visualize and analyse the chemical space of molecular datasets is the principal component analysis (PCA) with an appropriate set of molecular descriptors. Thus, the combination of the 7 discriminative descriptors cited above (molecular weight, AlogP, aromatic ratio and the 4 shape descriptors uncorrelated with neither the size nor the hydrophobicity) with 13 commonly used descriptors to depict chemical space (Table 2) provides us a way to depict the joined chemical space comprising all of the datasets. Moreover, in this chemical space, iPPI compounds can be characterized and distinguished from non-iPPI compounds. We therefore performed a principal component analysis on all of the cumulated datasets with those 20 descriptors (Fig. 4), representing a total of 569,456 molecules and 20 descriptors (See Methods). From Fig. 4, both iPPI-DB and TIMBAL compounds clearly show a nice global overlap between the two iPPI datasets (coloured dots and coloured squares in Panel A). Even though the two iPPI datasets can have different PPI targets, their corresponding iPPI compounds seem to overlap nicely as a whole. This trend is particularly noted when comparing the PPI targets that have iPPI compounds in both of the PPI datasets (but no duplicates), such as Bcl2, MDM2, Xiap, Brd, and IL2. For example, Xiap compounds (green dots and green squares) from both iPPI-DB and TIMBAL overlapped well in the individual map of Fig. 4 (Panel A). However, within the chemical space region corresponding to iPPI compounds, the compounds of different PPI targets interestingly occupy different regions. It is visible that iPPIs of Xiap (green), MLL (yellow), K-RAS (grey), and IL2 (orange) occupy the top of the individual map in Panel A, which, according to the circle of correlation (Panel B), defines a higher number of chiral centres and sp3 carbon atoms. In contrast, iPPIs of CD80 (in red) and of CTNNB1 (in beige) are at the bottom right corner of the individual map (Panel A), illustrating their higher proportion of aromatic atoms according to the same circle of correlation. The example given in Fig. 4 also shows the inhibitors of GPCRs (black dots). It is clear from the individual map (Panel A) that iPPIs (all of the non-black dots and squares) and GPCR inhibitors (black dots) occupy different regions in chemical space. Interestingly the most important chemical space imbalance comes from the first component of the PCA, which is mostly associated with EDmin3, confirming that iPPIs clearly have lower values than do non-iPPI compounds for that property.

### Evaluation of the imbalance in the iPPI chemical space

**Figure 5:**
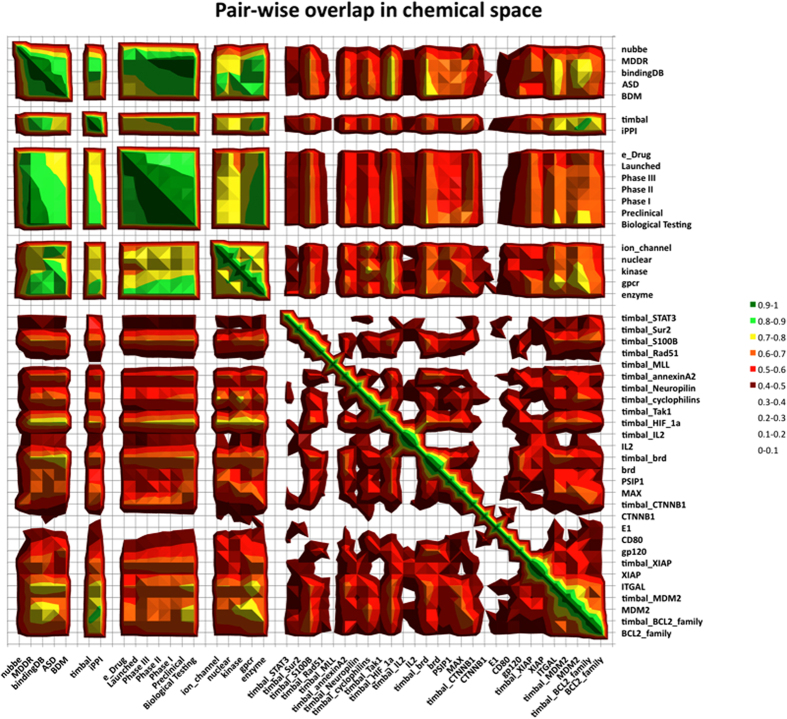
Surface map of the pairwise imbalance in chemical space of all of the datasets. In the top left corner are the Nubbe, MDDR, BindingDB, ASD, BDM, TIMBAL, and iPPI-DB datasets. The rest of the map contains the sub-datasets corresponding to MDDR (biological testing, preclinical, Phase I/II/III, and launched), e_Drug, BindingDB (ion channel, nuclear receptors, kinases, GPCR, and enzyme), and the individual PPI targets from both TIMBAL and iPPI-DB. Surface peaks are coloured based on the pairwise overlap in chemical space for two given populations. Overlap scores are between 0 and 1, such that according to the colour code, green regions demonstrate higher overlap, and red or even transparent regions demonstrate low overlap, if any, and therefore greater imbalance.

It is clear from the analysis of the PCA that interesting comments can be made to evaluate the qualitative imbalance in chemical space between datasets to link it to the principal components and therefore to the descriptors/properties that are responsible for this imbalance. Nevertheless, given the multiple dimensions of the PCA and the number of datasets (29 PPI targets when iPPI-DB and TIMBAL are combined and 17 non-iPPI datasets), it would be impossible to account for the imbalance between the datasets just by plotting their corresponding compounds into the successive components of the PCA, nor would it be sufficient to quantify it. To address this issue and provide ourselves with a procedure to actually quantify the imbalance in chemical space between the different datasets, we designed a simple metric relying on the principal components of a PCA. This procedure relies on the probability density functions of each dataset along each component of the PCA (see Methods). For each possible pair of datasets and each successive PCA component, the individual overlap δ (Equation 5 in Methods) between the two corresponding probability density functions was calculated. These one-component overlaps δ were then combined into an arithmetic mean Δ weighted by the variance associated with the corresponding component (see Equation 6 in Methods). The idea is to put more weight for the component overlap δ corresponding to the components carrying most of the variance. Collectively, this metric, in the form of a normalized score between 0 and 1, with 1 being complete overlap, quantifies the imbalance in chemical space for each possible pair of datasets in accounting for all of the components of the PCA. The results of such a measure can then be displayed as an array of pairwise normalized overlap scores for all possible pairs of datasets and colour-coded according to the values of their overlap score, as shown in the surface map of Fig. 5.

To assess the pertinence of such a metric, several observations can be made. First, the observed common region of chemical space shared by the iPPI-DB and the TIMBAL datasets depicted in Fig. 4 was confirmed by an overlap score Δ of 0.88. Second, commercial chemical libraries are poorly adapted for identifying modulators of PPIs because they have been designed for the modulation of more conventional targets, such as GPCRs, enzymes, and, more recently, kinases, nuclear receptors, and ion channels. This trend was confirmed by the overlap metric Δ between BDM, which is a representative dataset of three chemical compound providers, and the subsets GPCR, enzyme, kinase, nuclear receptors, ion channels, or the full MDDR dataset. Indeed, overlap scores Δ between BDM and these datasets were most often close to 0.85, while they were equal to 0.74 and 0.76 for the pairs (BDM, iPPI-DB) and (BDM, TIMBAL), respectively.

Third, the actual drugs and compounds in current drug development, such as those of e_Drug, launched (MDDR), phase I/II/III (MDDR), preclinical (MDDR) and biological testing (MDDR), are optimized to fit as much as possible a certain physicochemical profile in terms of size, complexity and hydrophobicity. This profile maximizes the chances of eventual success and might avoid a series of pharmacokinetic issues during development. Moreover, the present study shows that the 4 shape properties EDmin3, IW4, glob and CW2 were significantly different on average between those drug datasets and the two iPPI datasets (iPPI-DB and TIMBAL). These properties were represented among the 20 descriptors that were used to calculate the PCA and therefore were accounted for in the overlapping scores. It is therefore not surprising to picture these datasets as close in chemical space. In fact, it is clearly visible from the surface map of Fig. 5 that the overlap scores Δ of these datasets correspond to the largest values of overlap (largest green square), with overlap scores Δ greater than 0.9. Not only do these datasets seem to be characterized by the above-mentioned physicochemical profile, but they also seem to be equally dissimilar in chemical space to iPPI compounds regarding their shape properties. These points collectively justify the global overlap between these datasets and the selection of those descriptors.

Finally, it is interesting to note that enzymes and GPCRs have a higher overlap score Δ with respect to advanced phases of development in MDDR (Phases I, II, and III) than do kinases, nuclear receptors, and ion channels. This result again confirms a well-established fact that most of the developed drugs still aim at GPCRs or enzymes.

Having confirmed a series of pertinent observations, we then attempted to use the overlap metric Δ to identify new trends in this chemical space. The most striking observation concerns the heterogeneity of the PPI targets. Indeed, a rapid overlook of all of the individual PPI targets from both of the iPPI datasets (right lower quarter of the figure) is sufficient to perceive that they do not share the same level of overlap as the GPCR, enzyme, kinase, ion channel, and nuclear receptor datasets. We have already identified such heterogeneity in a previous study using a rather different approach^31^, and the need to address this heterogeneity by considering subclasses of PPI targets has also been underlined by others^32^. Furthermore, we can confirm some of the observations that we made simply using the individual map of Fig. 4 (Panel A). Indeed, PPI targets that have iPPI compounds in both iPPI-DB and TIMBAL (but no duplicates) have clearly higher overlap values: (Bcl2_family, timbal_Bcl2_family, Δ = 0.80), (MDM2, timbal_MDM2, Δ = 0.77), (Xiap, timbal_Xiap, Δ = 0.80), (Brd, timbal_Brd, Δ = 0.80), and (IL2, timbal_IL2, Δ = 0.81). In contrast, the overlap scores also distinguish iPPI datasets belonging to different regions of chemical space. The values of the overlap scores are rather different when considering the pair Xiap, IL2 (Δ = 0.54), which belongs graphically to the same region of the individual map of Fig. 4, or when considering the pair Xiap, CD80 (Δ = 0.31) which belongs to rather different regions. Furthermore, the surface map of Fig. 5 also shows that PPI targets, such as HIF-1A, have a higher overlap score Δ with non-iPPI datasets than with other PPI targets. Finally, PPI targets, such as CTNNB1, timbal_MLL, or E1, are clearly described as outliers with respect to the rest of the targets (PPI or not).

### Defining homogenous regions of the iPPI chemical space

**Figure 6:**
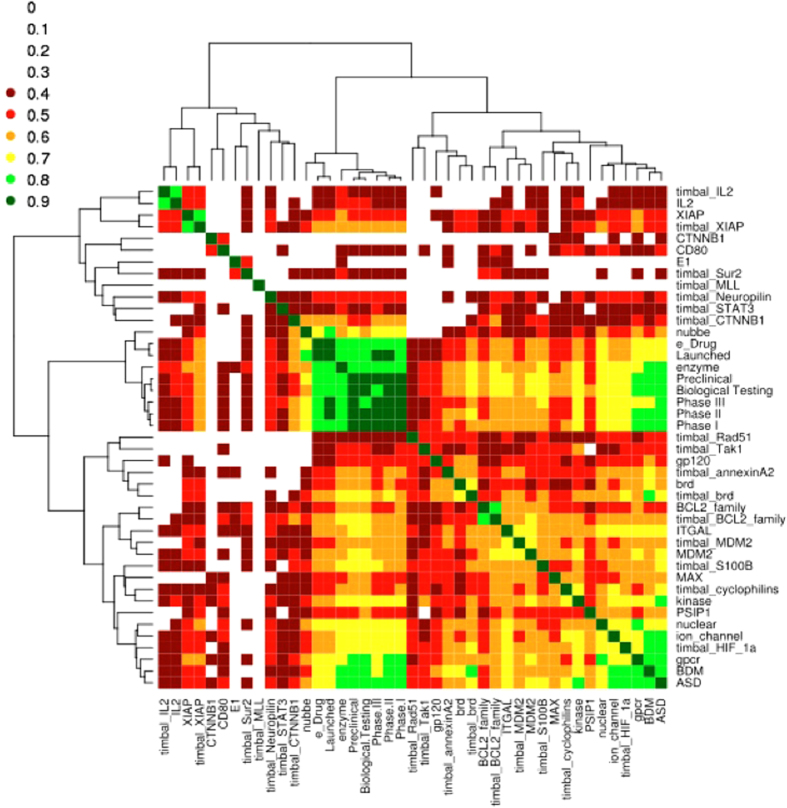
Heat map and dendrogram resulting from an agglomerative hierarchical classification of all of the datasets based on the overlap in chemical space. The green regions represent higher overlap, the red regions demonstrate moderate overlap, and the transparent regions represent low overlap.

The surface map of Fig. 5 could highlight the global positioning of each PPI target with respect to the remaining PPI targets, and that classes of PPI targets could be identified using the same metric. To confirm those observations, we then used the metric described herein as a distance criterion and proceeded to an agglomerative hierarchical clustering of all of the datasets using the Ward method^33^. The clustering was combined with a heat map (see Methods) to visualize the different classes and gauge the internal homogeneity of the resulting clusters (Fig. 6). To prevent redundancy and any bias of the results, we only kept sub-datasets. Indeed, we kept all of the PPI targets individually but removed iPPI-DB and TIMBAL as a whole. We proceeded to the same operation for MDDR and BindingDB by just keeping the sub-datasets that comprised them. Except for the datasets that were removed to prevent redundancy, the same array of pairwise overlap scores Δ that was used to make Fig. 5 was also used to feed the agglomerative hierarchical clustering using the Ward method.

As expected, clustering gathers all of the subsets of MDDR (biological testing, preclinical, phase I/II/III, launched), e_Drug, Nubbe and enzyme active compounds. Indeed, the natural compounds of Nubbe appear to be close to MDDR compounds. Similarly, active compounds on enzymes are closer to MDDR compounds because they still account for the majority of the existing active compounds and drug candidates.

Clustering showed a strong overlap between nuclear receptors, GPCRs, ion channel, BDM, ASD, and timbal_HIF-1A, with an overlap score Δ greater than 0.7 on average. This result confirms the observation made on the surface map from Fig. 5 that this PPI target is closer to non-iPPI datasets than to other PPI targets. It is worth noting that these datasets have also an important overlap with MDDR subsets. Finally, these results confirm that commercial libraries, such as BDM (~40,000 from three providers), are closer to active compounds on conventional targets than to iPPI. Similarly, allosteric compounds from ASD are also closer to commercial databases and GPCR compounds, which account for a substantial number of allosteric compounds, than inhibitors of protein-protein interactions.

However, most interestingly, the results show a clear separation between inhibitors of most PPI targets from non-iPPI datasets and, more convincingly, of the corresponding PPI targets among themselves. Indeed, the conclusive aspects of clustering are the creation of classes of PPI targets, whose iPPI compounds seem to share physicochemical profiles. One can note the proximities of the following PPI targets: (IL2 and Xiap), (CD80 and CTNNB1), (Brd, annexinA2, and GP120), (MDM2, ITGAL and, to a lesser extent, BCL2). This pattern also confirms that iPPIs of a given PPI target from both iPPI-DB and TIMBAL, such as (timbal_IL2, IL2), (timbal_XIAP, XIAP), (timbal_brd, brd), and (timbal_MDM2, MDM2), most often fall into a highly homogeneous class (Δ~0.8). Therefore, such an approach allows the definition of homogenous regions in the iPPI chemical space. There are also PPI targets that are close to non-iPPI datasets, such as PSIP1 with BindingDB subsets; S100B, MAX, and cyclophilin with kinase; and timbal_HIF-1A as mentioned above.

### Pocket-driven evaluation of the PPI target space

To further measure the heterogeneity of the PPI target space and to confirm some of the groups of PPI targets mentioned above, we used a purely pocket-driven approach without relying on the physicochemical properties of the iPPI compounds. To this end, we proceeded to analyse the pocket properties of all of the PPI targets that were present in both TIMBAL and iPPI-DB and whose experimental structures (X-ray crystallography or NMR) were available in the PDB.

Using the programs VolSite^12^ and MOE^34^, we detected the binding pockets of the PPI targets and calculated a series of 117 pocket descriptors: 89 from VolSite, 10 using a combination of VolSite descriptors and 18 calculated with MOE on the negative image of the binding pockets provided by VolSite (see Methods). These descriptors were then used to calculate the Euclidean distance between pockets and to carry out a clustering (Fig. S6) of all of the available crystal structures of the PPI target binding pockets from the datasets using an agglomerative hierarchical clustering with the Ward method (see Methods).

From the clustering, it is clear that most of the structures of a given PPI target are grouped together when considering 4 clusters, with the exception of Bcl-2, which falls into two distinct groups. This target has a very large pocket that binds the BH3 domain of its partners, consisting of an 18-amino-acid-long α-helix lining the groove of Bcl-2. The present pocket is highly flexible and accommodates according to the nature of the BH3-only partners that it binds^35^. This result may explain the separation of the BCL-2 structures into two groups. Moreover, all of the structures of a given PPI target fall into the same cluster regardless of whether the experimental structures were resolved with or without a ligand (holo- or apo-form of the protein). However, more importantly, classes of PPI targets could be identified, some confirming the results of the ligand-based clustering approach that was described in the previous paragraph. Indeed, clustering unambiguously provides similar results to that carried out with the iPPIs alone. Thus, when considering 4 clusters, Brd is grouped with GP120, MDM2 is grouped with ITGAL and partially with Bcl-2, Xiap is grouped with IL-2, and the rest of the Bcl-2 structures are grouped together. It is worth noting that PSIP1 is now grouped with MDM2 and ITGAL but mostly with non-iPPI datasets before in the ligand analysis.

In order to characterize each of these 4 groups of targets, we selected the corresponding structures that clustered within each of these groups and calculated an ANOVA followed by post hoc comparisons using a pairwise test between groups and for each pocket descriptor. This process has allowed us to determine which pocket descriptors were significantly different (p-value < 1.10^−4^) for each group with respect to the 3 remaining groups and provides a physicochemical profile of a representative pocket for each of the 4 groups. Thus, the group of Brd and GP120 is characterized by more globular and more buried pockets with more hydrogen bond acceptors. MDM2 and ITGAL are more aromatic, less hydrophobic, moderately buried, and with fewer hydrogen bond acceptors and fewer hydrogen acceptors and donors combined. Xiap and IL-2 are smaller and more exposed (shallow), rod-like with more hydrogen bond donors and with more positive and negative charges. Bcl-2 pocket are larger, more hydrophobic with negative charges, less aromatic, less globular and with fewer hydrogen bond donors.

### Crossing the PPI chemical and target spaces

**Figure 7:**
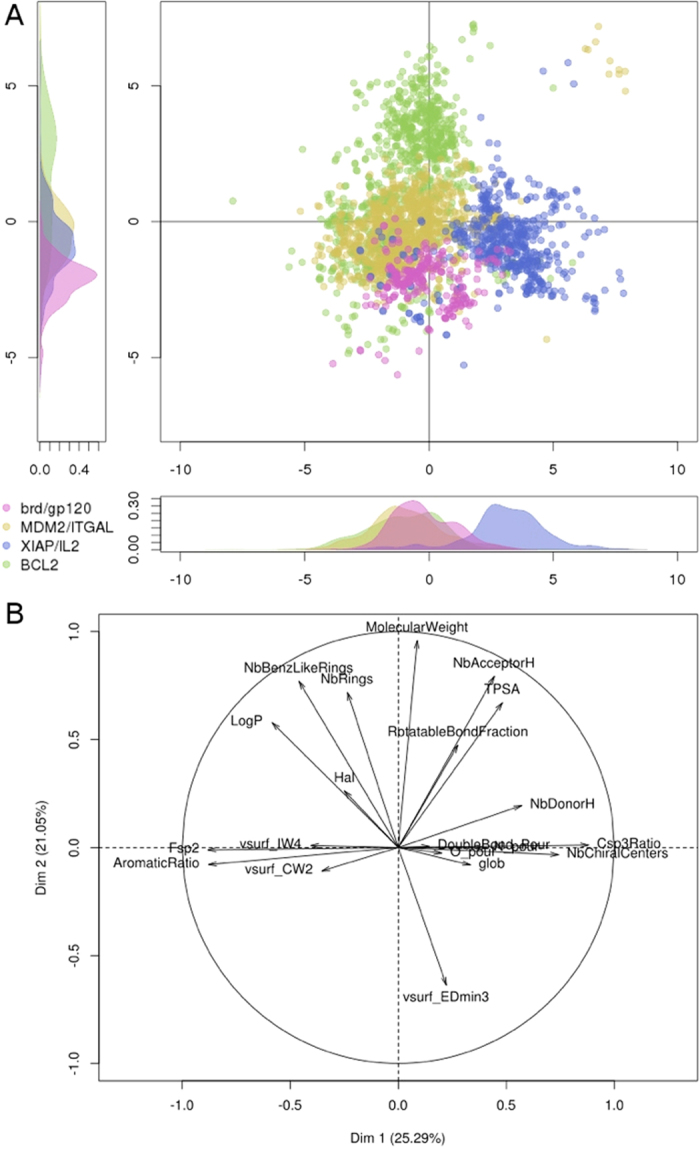
PCA representing the iPPI compounds of the 4 groups of PPI targets. Group 1 (Brd and GP120) as pink, Group 2 (MDM2 and ITGAL) as yellow, Group 3 (Xiap and IL2) as blue, and Group 4 (Bcl2) as green. (**A**) Individual map of the compounds and density curves on each axis for the 4 populations from each of the 4 groups. (**B**) Variable map, circle of correlation.

The fact that some PPI targets fall into the same classes using two orthogonal methods, such as pocket-based and ligand-based approaches, led us to retrospectively inspect which regions of the chemical space these groups correspond to. Thus, we decided to closely inspect the regions of chemical space enclosing the iPPI compounds of GP120, Brd, MDM2, ITGAL, Xiap, IL-2 and Bcl-2. To do so, we plotted an individual map of the PCA, including all of the corresponding iPPI compounds, by colour coding the above-mentioned groups, i.e., Group 1 (Brd with GP120), Group 2 (MDM2 with ITGAL), Group 3 (Xiap with IL-2) and Group 4 (Bcl-2) (Fig. 7). From the PCA individual map, one can note that, even for the two first components (46% of the total variance), Groups 1, 2, and 3 can be easily separated. Group 4 partially overlaps with Group 2, confirming the results of the pocket-driven clustering that Bcl-2 and both MDM2 and ITGAL share a region for their chemical and target spaces. This figure nicely confirms that such clustering can be performed independently and cohesively using iPPI compounds or PPI pocket descriptors. Not only do some of the PPI pockets seem to share properties, but these properties seem to condition the physicochemical properties of their respective ligand. More precisely, the perspective to further identify homogenous regions of the PPI target space corresponding to homogenous regions of the iPPI chemical space is key to designing focused chemical libraries that are dedicated to classes of PPI binding cavities. This process would become particularly efficient when combined with properties such as EDmin3, which manages to differentiate more generally an iPPI compound from a conventional molecule regardless of the PPI target.

**Figure 8:**
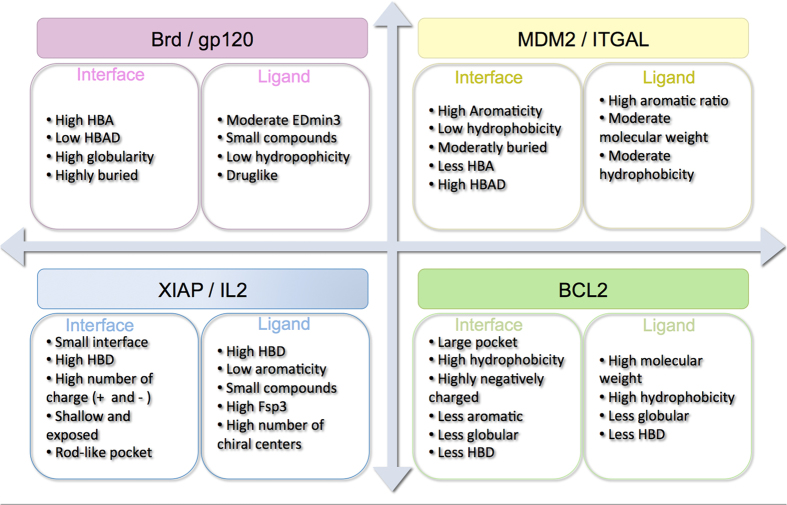
Representation of the important properties for each cluster of the left pocket properties and on the right ligand properties. HBD: donors of hydrogen bonds, HBA: acceptors of hydrogen bonds, HBAD: acceptors and donors of hydrogen bonds, Fsp3: ratio of Csp3 carbons.

To inspect the properties of the shared regions of the chemical space and of the target space, we summarized the properties of both the iPPI compounds and the PPI targets corresponding to the PPI groups mentioned above (Fig. 8). This summary demonstrates that homogenous and cohesive regions of chemical and target space can be found and that these identified groups of targets could lead to the design of PPI-class-specific chemical libraries.

## Conclusion

Fundamental processes in living cells are largely controlled by protein-protein interactions. The deregulation of these interactions plays a critical role in the pathogenesis of numerous diseases. Thus, PPIs represent attractive targets even though targeting them with LMW compounds still represents a major challenge. The aim of this analysis was to capitalize on existing knowledge of the iPPI chemical space and to gauge the heterogeneity of the PPI target space. During this analysis, we confirmed that iPPIs present a specific profile according to their size, hydrophobicity, aromaticity, capacity to bind hydrophobic patches (EDmin3), globularity (glob), percentage of exposed polar groups (CW2), and location of these polar groups (IW4). Interestingly, we could distinguish properties that seem to apply to nearly all of the PPI targets, such as a higher molecular weight or the shape property EDmin3, and properties that seem to be more specific to some PPI families, such as logP, aromatic ratio, globularity, CW2 and IW4. These results highlight that more effort should be put into designing or selecting compounds that have hydrophobic chemical moieties at the right location rather than being on average simply more hydrophobic. A proper layout can be easily evaluated with descriptors, such as EDmin3. These results also confirm some discrepancies among PPI targets. The metric Δ is presented to quantify the overlap in chemical space between two molecular datasets using PCA calculated with some of the iPPI-specific descriptors cited above. The use of this metric has not only confirmed a high discrepancy among the different PPI targets, but more importantly has highlighted classes of PPI targets according to the profile of their iPPI compounds. Most interestingly, when using an orthogonal analysis based on a purely pocket-driven approach, some of the most important classes of PPI targets could be confirmed, demonstrating that homogenous regions of the iPPI chemical space can be affixed to homogenous regions of the PPI target space.

We anticipate new types of PPI pockets in the near future that will either merge or supplement those described herein. However, given the profiles that are presented in this study, we might speculate that any given PPI pocket characterized by similar pocket properties may require iPPIs with similar properties. Moreover, the fact that we managed to partially propose the independent and cohesive clustering of the PPI targets regardless of whether they are processed using iPPI compounds or pocket descriptors represents a proof-of-concept that homogenous regions of the PPI chemical space can be identified and be coherent with homogenous regions of the PPI target space. This proof of concept is therefore independent of the methods used herein, and we anticipate that new methodological developments will support this strategy because not only do the PPI pockets seem to share properties, but they seem to condition the physicochemical properties of their respective ligands. This characteristic will be a strong factor in further developing such approaches in the near future and promulgating PDB-wide pocket analysis. Such large data analyses could provide essential insights into the feasibility of a given project based on the proximity of the PPI target of interest with previously chemically probed interfaces. In turn, these results could help to prioritize the choice of PPI targets based not only on cellular mechanisms or the druggability of their interfaces, but also on the chemical risks that one may encounter by investigating it. This information will certainly assist in the preparation of dedicated compound libraries tuned for classes of homogenous PPI targets and will in turn facilitate the identification of protein-protein interaction inhibitors.

## Methods

### Dataset compilation

#### Inhibitors

A dataset of iPPIs was constructed from iPPI-DB (version 2013)^13^ (1,650 compounds) and TIMBAL (version May 2015)^3^ (8,107 compounds). In order to select the more accurate dataset, several criteria were applied. The information about the PPI had to be unambiguous. The modulation had to be inhibition and not stabilization. We selected only those compounds with the following measures of activity: K_d_, K_i_, IC_50_ and EC_50_. Moreover, this activity had to be less than 30 μM. We excluded small metal-based compounds, peptides, macrocycles and molecules containing atoms different from C, N, O, S, P and halogens. Finally a set of 3,248 iPPIs was constructed: 1,650 from iPPI-DB and 1,598 from TIMBAL.

We also constructed different datasets of so-called non-iPPIs from different sources. In this study, non-iPPIs are molecules that do not inhibit protein-protein interfaces: inhibitors of conventional targets, such as GPCR or enzymes; known drugs; allosteric compounds; natural products; compounds in preclinical or clinical phases; or compounds from commercial databases. The description and number of compounds in each dataset are described in Table 1.

For all of the compounds (iPPIs and non-iPPIs), we removed redundant compounds, peptide molecules defined as compounds with more than 3 contiguous peptide bonds, salts, compounds with less than 10 heavy atoms, carbocations, and molecules containing atoms different from C, N, O, S, P and halogens. Only compounds with a molecular weight of less than 1,200 ^g.mol−1^ were selected. All of the compounds were normalized and standardized with PipelinePilot (v9.0.2) using the same standardization protocol.

#### Interfaces

A total of 62 protein-protein interfaces were collected from the PDB^36^ or 2P2I-DB^30^ and referred to the PPI complexes in iPPI-DB or TIMBAL. A total of 84 inhibitor-protein complexes (from PPI) were collected from the PDB and 2P2I-DB^37^. These complexes were found across 15 different PPI families.

### Molecular descriptors

#### Inhibitors

A set of 16 molecular 2D descriptors was calculated in this analysis. These descriptors were calculated with a java program using ChemAxon JChem library (version 5.10.1, https://www.chemaxon.com/). These descriptors were a set of classical descriptors and are described in Table 2.

Another set of 3D descriptors (vsurf_EDmin3, vsurf_CW2, vsurf_IW4 and glob) was also calculated using Moe software (version 2012.10) using a previously described protocol^11^.

#### Interfaces

A set of 89 descriptors for the interface were calculated using VolSite software^38^ (version 2014). The different parameters for all of the different interfaces were the minimal number of cubes required to consider it as a cavity equal to 20, the edge length of the main box equal to 20 Å, the minimal number of neighbours for buried cavity boxes equal to 7, and the edge length of each box equal to 1.5 Å, and hydrogen atoms were considered. For the PPI interfaces, the minimal threshold for buriedness was set to 50 and 60 for the non-PPI interfaces.

All of the different interfaces were manually inspected to ensure that the detected pocket was at the right location on the protein surface.

For all of the pockets that were divided into different sub-pockets, different steps were used to combine them and obtain descriptors for a global pocket. The cavity volume was obtained by adding the cavity volume of different sub-pockets (see Equation 1 for two sub-pockets).


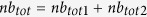


Equation 1: The calculation of the total number of points present in the combined pocket with nb_tot1_ as the total number of points present in sub-pocket 1 and with nb_tot2_ as the total number of points present in sub-pocket 2.

The number of points of each feature for each pocket (see Equation for an example: hydrophobic points) divided by the total number of points was calculated by to evaluate the global percentage (see Equation 3) for an example: hydrophobic points) of each feature (hydrophobic, aromatic, H-bond acceptor, negative ionizable, H-bond acceptor/donor, H-bond donor, positive ionizable and dummy atoms)


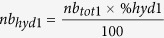


Equation 2: Calculation of the number of hydrophobic points present in sub-pocket 1, with %hyd1 as the percent of hydrophobic points in the sub-pocket.


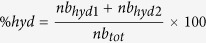


Equation 3: Calculation of the percent of hydrophobic points in the global pocket.

Using the number of each feature point for each pocket at a specific distance (see Equation 4 for an example: pocket 1 and hydrophobic point at 40–50), the percentage of each feature with a projection value between 2 distances was obtained with Equation 5 (for example: the hydrophobic points and distance between 40 and 50)


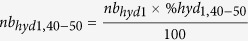


Equation 4: Calculation of the number of hydrophobic points in sub-pocket 1 between 40 and 50.


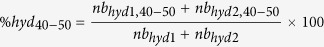


Equation 5: Calculation of the percent of hydrophobic points between 40 and 50 for the global pocket.

For all of the evaluated pockets, 10 descriptors were added for this analysis. These descriptors were the sum of the different features (hydrophobic, aromatic, H-bond acceptor, negative ionizable, H-bond acceptor/donor, H-bond donor, positive ionizable and dummy atoms) at each distance (Equation 6).


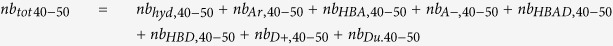


Equation 6: Calculation of the combination of descriptors at each distance.

For each evaluated pocket, VolSurf provides a mol2 file with all of the different probes. This file was used as a template in Moe (version 2012.10), and the 18 descriptors that are present in Table 3 were calculated.

Finally, a set of 117 descriptors (89 from VolSite, 10 from a combination at each distance and 18 from Moe) was calculated on the different interfaces.

### Ligand analysis

#### PAINS/ADME-Tox filter

PAINS (pan assay interference compounds) (filters A, B and C) and ADME-Tox filter (Table 4) were calculated using the online program FAFDrugs 3^23^ (http://fafdrugs3.mti.univ-paris-diderot.fr/). All of the parameters were set to default values.

#### Molecular descriptors analysis

All of the multivariate or univariate analyses were performed using scripts from the software package R (version 2.15.1). Graphical analyses were performed using R (version 2.15.1) or Excel (Mac 2008). A PCA was performed using the FactoMineR library, which scaled the input data prior to the analysis. The probability density functions on the PCA were calculated based on the successive principal components with the R module Density set to default parameters. Heat maps were calculated using either with heat map packages using R or with the graphics type “surface” in Excel, and dendrograms on the heat maps were implemented with the Ward method^33^ using Euclidean distances.

#### Analysis of the discrimination between two populations

For all of the different statistical tests, only families with five or more compounds were analysed^39^. Thus, out of the 39 PPI families (13 from iPPI-DB and 26 from TIMBAL), only 28 PPI families were analysed.

The two datasets were compared using Student’s test if the two datasets followed a normal distribution according to the Shapiro test and had equivalent variances according to the Fisher–Student test.

If the previous conditions were not fulfilled, comparisons were made using the nonparametric Mann–Witney–Wilcoxon test. The population mean values were compared using a nonparametric Kruskal–Wallis ANOVA. Post hoc comparisons were carried out using a pairwise Mann–Whitney–Wilcoxon test^40^.

For all of the different comparisons, the statistical tests were two-tailed with an alpha equal to 0.05 and were considered significant when the p-value was less than 0.05.

#### Determination of the overlap between two populations

First, a principal component analysis was calculated using R and the FactoMineR package on all of the different datasets (Table 1). Then, the coordinates from the PCA were extracted for the two considered datasets (see example in Fig. S7).

On each component of the PCA and for each of the two datasets, the probability density function was calculated using the R density module set to default parameters. The overlap on one component between two datasets was the area in common between the two densities (in violet in the Fig. S7). To calculate this overlap δ on one component between two datasets, we used Weitzman’s Measure (Equation 7)^41^.


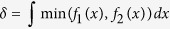


Equation 7: Weitzman’s Measure^41^ with f_1_ as the density for the first dataset and f_2_ as the density for the second dataset.

Then, to combine the overlap for all of the components, Equation 8 was used. This combination is an arithmetic mean weighted by the variance of each component (to reach 100% of the cumulated variance). Finally, an overlap score Δ for each pair of datasets was obtained. This score was between 0 and 1, with 0 meaning completely dissimilar datasets and 1 meaning identical datasets. These operations were performed for all of the datasets versus all of the components to obtain an N×N array of Δ scores.


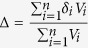


Equation 8: Weighted arithmetic mean with δ_i_ is the overlap between the two densities for the axis i from the PCA, Vi is equal to the variance percentage for the axis i from the PCA and n is the number of dimensions to reach 100% of the variance.

### Interface analysis

#### Analysis of Hclust

Clusters were calculated on the different binding pockets using Euclidian distances and the Ward method within the Hclust R package. A comparison test was performed on each different group of the clustering. The corresponding structures of each group were selected, and an ANOVA was calculated followed by post hoc comparisons using a pairwise test between groups. The population mean values were compared using a nonparametric Kruskal–Wallis ANOVA. Post hoc comparisons were carried out using a pairwise Mann–Whitney–Wilcoxon test^40^. For all of the different comparisons, statistical tests were two-tailed with an alpha equal to 1E-04 and were considered significant when the p-value was less than 1E-04. Therefore, only very significant discriminating descriptors were selected to describe each group.

## Additional Information

**How to cite this article**: Kuenemann, M.A. *et al*. Imbalance in chemical space: How to facilitate the identification of protein-protein interaction inhibitors. *Sci. Rep.* **6**, 23815; doi: 10.1038/srep23815 (2016).

## Supplementary Material

Supplementary Information

## Figures and Tables

**Table 1:** Dataset descriptions for the non-iPPI datasets.

Type	Name	Size	Description
Compounds in biological testing, preclinical or clinical phases	MDDR complete v	222,567	All of the compounds in biological testing, preclinical and clinical trials
	Biological Testing (MDDR subset)	202,443	Compounds in biological testing
	Preclinical (MDDR subset)	15,522	Compounds in preclinical trials
	Phase I (MDDR subset)	965	Compounds in Phase I
	Phase II (MDDR subset)	1198	Compounds in Phase II
	Phase III (MDDR subset)	304	Compounds in Phase III
	Launched (MDDR subset)	1420	Compounds already on the market (FDA approved)
Drug orally bioavailable	E_Drugs v^16^	916	Orally bioavailable FDA-approved compounds
Allosteric compounds	Allosteric v^17^	14,873	Allosteric modulators
Inhibitors of conventional targets	Binding DB v^18^	44,241	Compounds modulating the subset of the 100 most represented targets
	Enzyme (subset of BindingDB)	17,335	Compounds modulating (non-kinase) enzymes
	Kinase (subset of Binding DB)	4132	Compounds modulating kinases
	Gpcr (subset of Binding DB)	19,952	Compounds modulating GPCR
	Ion Channel (subset of Binding DB)	725	Compounds modulating ion channels
	Nuclear (subset of Binding DB)	2097	Compounds modulating nuclear receptors
Natural compounds	Nubbe v^22^	617	Natural products and derivatives from Brazilian biodiversity
Compounds from commercial databases	BDM	39,186	Compounds from commercial databases (Asinex, ChemDiv and Enamine)

**Table 2:** All of the names of the 2D descriptors and their description.

Name	Description
Fsp2	Number of carbon sp2 divided by the number of carbons
Aromatic Ratio	Number of aromatic rings divided by the number of heavy atoms
Molecular Weight	Molecular weight of the compound
LogP	ALogP
TPSA	Topological surface area
Nb Acceptor H	Number of atom acceptors of hydrogen
Nb Donor H	Number of atom donors of hydrogen
Nb Chiral Centers	Number of chiral centres
Rotatable Bond Fraction	Number of rotatable bonds divided by the number of heavy atoms
Nb Rings	Number of rings
Nb Benz Like Rings	Number of benzene-like rings
Csp3 Ratio	Number of carbon sp3 divided by the number of carbons
O_pour	Number of oxygen divided by the number of heavy atoms
N_pour	Number of nitrogen divided by the number of heavy atoms
Double Bond_Pour	Number of doubled bonds divided by the number of bonds
Hal	Number of halogens
Vsurf_EDmin3	Local interaction minimum
Vsurf_IW4	Hydrophilic interaction energy moment at −2 kcal/mol
Vsurf_CW2	Capacity factor at −0.5 kcal/mol
Glob	Globularity

**Table 3:** Pocket descriptors calculated by Moe.

Descriptors Name	Description
Glob	Molecular globularity
npr1	Normalized PMI ratio (1) (pmi1/pmi3)
npr2	Normalized PMI ratio (2) (pmi2/pmi3)
pmi	Principal moment of inertia
pmi1	Principal moment of inertia (1)
pmi2	Principal moment of inertia (2)
pmi3	Principal moment of inertia (3)
pmiX	Principal moment of inertia (X)
pmiY	Principal moment of inertia (Y)
pmiZ	Principal moment of inertia (Z)
rgyr	Radius of gyration
std_dim1	Standard dimension 1
std_dim2	Standard dimension 2
std_dim3	Standard dimension 3
vol	Van Der Waals volume
VSA	Van Der Waals surface area
Vsurf_G	Surface globularity
Vsurf_R	Surface rugosity

**Table 4:** ADME-tox filter used on FAF-Drug3, names and descriptions.

Filter	Explanation
Lipinski rule of five^25^	An oral bioavailability evaluation
Veber^42^	An oral bioavailability evaluation
Egan^42^	An oral bioavailability evaluation
GSK’s 4/400^28^	A drug safety profiling
Pfizer’s 3/75^43^	A drug safety profiling
Lilly MedChem Rules^27^	Identify compounds that may interfere with biology

## References

[b1] Wells J. A. & Mcclendon C. L. Reaching for high-hanging fruit in drug discovery at protein-protein interfaces. Nature 450, 1001–1009 (2007). 18075579 10.1038/nature06526

[b2] Sperandio O., Reynès C. H., Camproux A.-C. & Villoutreix B. O. Rationalizing the chemical space of protein-protein interaction inhibitors. Drug discovery today 15, 220–229 (2010). 19969101 10.1016/j.drudis.2009.11.007

[b3] Higueruelo A. P. . Atomic interactions and profile of small molecules disrupting protein-protein interfaces: the TIMBAL database. Chemical biology & drug design 74, 457–467 (2009). 19811506 10.1111/j.1747-0285.2009.00889.x

[b4] Morelli X., Bourgeas R. & Roche P. Chemical and structural lessons from recent successes in protein-protein interaction inhibition (2P2I). Current Opinion in Chemical Biology 15, 475–481 (2011). 21684802 10.1016/j.cbpa.2011.05.024

[b5] Hann M. M. Molecular obesity, potency and other addictions in drug discovery. Med Chem Comm 2, 349–355 (2011).

[b6] Ritchie T. J. & Macdonald S. J. The impact of aromatic ring count on compound developability--are too many aromatic rings a liability in drug design? Drug Discov Today 14, 1011–1020 (2009). 19729075 10.1016/j.drudis.2009.07.014

[b7] Ritchie T. J., Macdonald S. J., Young R. J. & Pickett S. D. The impact of aromatic ring count on compound developability: further insights by examining carbo- and hetero-aromatic and -aliphatic ring types. Drug Discov Today 16, 164–171 (2011). 21129497 10.1016/j.drudis.2010.11.014

[b8] Neugebauer A., Hartmann R. W. & Klein C. D. Prediction of protein-protein interaction inhibitors by chemoinformatics and machine learning methods. Journal of Medicinal Chemistry 50, 4665–4668 (2007). 17705363 10.1021/jm070533j

[b9] Reynès C. . Designing focused chemical libraries enriched in protein-protein interaction inhibitors using machine-learning methods. PLos computational biology 6, e1000695 (2010). 20221258 10.1371/journal.pcbi.1000695 PMC2832677

[b10] Fry D. . Design of Libraries Targeting Protein-Protein Interfaces. Chem Med Chem 8, 726–732 (2013). 23436619 10.1002/cmdc.201200540

[b11] Kuenemann M. A., Bourbon L. M., Labbe C. M., Villoutreix B. O. & Sperandio O. Which three-dimensional characteristics make efficient inhibitors of protein-protein interactions? J Chem Inf Model 54, 3067–3079 (2014). 25285479 10.1021/ci500487q

[b12] Cruciani G., Crivori P., Carrupt P.-A. & Testa B. Molecular fields in quantitative structure–permeation relationships: the VolSurf approach. Journal of Molecular Structure: THEOCHEM 503, 17–30 (2000).

[b13] Labbe C. M., Laconde G., Kuenemann M. A., Villoutreix B. O. & Sperandio O. iPPI-DB: a manually curated and interactive database of small non-peptide inhibitors of protein-protein interactions. Drug Discov Today 18, 958–968 (2013). 23688585 10.1016/j.drudis.2013.05.003

[b14] Higueruelo A. P., Jubb H. & Blundell T. L. TIMBAL v2: update of a database holding small molecules modulating protein-protein interactions. Database: the journal of biological databases and curation 2013, bat039-bat039 (2013). 10.1093/database/bat039 PMC3681332 23766369

[b15] Berman H. M. . The Protein Data Bank. Acta crystallographica. Section D, Biological crystallography 58, 899–907 (2002). 12037327 10.1107/s0907444902003451

[b16] Pihan E., Colliandre L., Guichou J. F. & Douguet D. e-Drug3D: 3D structure collections dedicated to drug repurposing and fragment-based drug design. Bioinformatics 28, 1540–1541 (2012). 22539672 10.1093/bioinformatics/bts186

[b17] Huang Z. . ASD: a comprehensive database of allosteric proteins and modulators. Nucleic Acids Res 39, D663–669 (2011). 21051350 10.1093/nar/gkq1022 PMC3013650

[b18] Liu T., Lin Y., Wen X., Jorissen R. N. & Gilson M. K. BindingDB: a web-accessible database of experimentally determined protein–ligand binding affinities. Nucleic acids research 35, D198–D201 (2007). 17145705 10.1093/nar/gkl999 PMC1751547

[b19] Chemical compound provider : Asinex (http://www.asinex.com, 101N Chestnut St # 104, Winston-Salem, NC 27101, USA; 2012). Accessed: 1st November 2012.

[b20] Chemical compound provider : ChemDiv (http://www.chemdiv.com, 6605 Nancy Ridge Drive San Diego, CA 92121, USA; 2012). Accessed: 1st November 2012.

[b21] Chemical compound provider : Enamine (http://www.enamine.net, Enamine LLC, Princeton Corporate Plaza, 7 Deer Park Drive, Ste. M-3, Monmouth Jct., NJ 08852, USA; 2012). Accessed: 1st November 2012.

[b22] Valli M. . Development of a natural products database from the biodiversity of Brazil. J Nat Prod 76, 439–444 (2013). 23330984 10.1021/np3006875

[b23] Lagorce D., Sperandio O., Baell J. B., Miteva M. A. & Villoutreix B. O. FAF-Drugs3: a web server for compound property calculation and chemical library design. Nucleic Acids Res 43, W200–207 (2015). 25883137 10.1093/nar/gkv353 PMC4489254

[b24] Baell J. B. & Holloway G. A. New substructure filters for removal of pan assay interference compounds (PAINS) from screening libraries and for their exclusion in bioassays. J Med Chem 53, 2719–2740 (2010). 20131845 10.1021/jm901137j

[b25] Lipinski C. A., Lombardo F., Dominy B. W. & Feeney P. J. Experimental and computational approaches to estimate solubility and permeability in drug discovery and development settings. Advanced drug delivery reviews 46, 3–26 (2001). 11259830 10.1016/s0169-409x(00)00129-0

[b26] Veber D. F. . Molecular properties that influence the oral bioavailability of drug candidates. J Med Chem 45, 2615–2623 (2002). 12036371 10.1021/jm020017n

[b27] Bruns R. F. & Watson I. A. Rules for identifying potentially reactive or promiscuous compounds. J Med Chem 55, 9763–9772 (2012). 23061697 10.1021/jm301008n

[b28] Gleeson M. P. Generation of a set of simple, interpretable ADMET rules of thumb. J Med Chem 51, 817–834 (2008). 18232648 10.1021/jm701122q

[b29] Egan W. J., Merz K. M. Jr. & Baldwin J. J. Prediction of drug absorption using multivariate statistics. J Med Chem 43, 3867–3877 (2000). 11052792 10.1021/jm000292e

[b30] Hamon V. . 2P2IHUNTER: a tool for filtering orthosteric protein-protein interaction modulators via a dedicated support vector machine. Journal of The Royal Society Interface 11, 2013 0860–20130860 (2013). 10.1098/rsif.2013.0860 PMC3836326 24196694

[b31] Villoutreix B. O., Labbé C. M., Lagorce D., Laconde G. & Sperandio O. A leap into the chemical space of Protein-Protein Interaction inhibitors. Curr pharm des 18, 4648–4667 (2012). 22650260 10.2174/138161212802651571 PMC3901718

[b32] Mullard A. Protein–protein interaction inhibitors get into the groove. Nature Reviews Drug Discovery 11, 173–175 (2012). 10.1038/nrd3680 22378255

[b33] Ward J. H. Jr. Hierarchical grouping to optimize an objective function. Journal of the American Statistical Association 58, 236–244 (1963).

[b34] Molecular Operating Environment (MOE), 2013.08; Chemical Computing Group Inc., 1010 Sherbooke St. West, Suite #910, Montreal, QC, Canada, H3A 2R7, 2016. *Chemical Computing Group, Inc*.

[b35] Johnson D. K. & Karanicolas J. Druggable protein interaction sites are more predisposed to surface pocket formation than the rest of the protein surface. PLos computational biology 9, e1002951 (2013). 23505360 10.1371/journal.pcbi.1002951 PMC3591273

[b36] Berman H. M. . The Protein Data Bank. Nucleic Acids Res 28, 235–242 (2000). 10592235 10.1093/nar/28.1.235 PMC102472

[b37] Bourgeas R., Basse M.-J., Morelli X. & Roche P. Atomic analysis of protein-protein interfaces with known inhibitors: the 2P2I database. 5, e9598 (2010). 10.1371/journal.pone.0009598 PMC2834754 20231898

[b38] Desaphy J., Azdimousa K., Kellenberger E. & Rognan D. Comparison and druggability prediction of protein-ligand binding sites from pharmacophore-annotated cavity shapes. J Chem Inf Model 52, 2287–2299 (2012). 22834646 10.1021/ci300184x

[b39] Zhao Y. D., Rahardja D. & Qu Y. Sample size calculation for the Wilcoxon-Mann-Whitney test adjusting for ties. Statistics in medicine 27, 462–468 (2008). 17487941 10.1002/sim.2912

[b40] Bauer D. F. Construction confidence sets using rank statistics. Journal of the American Statistical Association 67, 687–690 (1972).

[b41] Weitzman M. S. Measures of overlap of income distributions of white and Negro families in the United States., Vol. Technical report 22. (U.S. Department of Commerce, Bureau of the Census, Washington, DC., 1970).

[b42] Veber D. F. . Molecular properties that influence the oral bioavailability of drug candidates. Journal of Medicinal Chemistry 45, 2615–2623 (2002). 12036371 10.1021/jm020017n

[b43] Hughes J. D. . Physiochemical drug properties associated with *in vivo* toxicological outcomes. Bioorganic & medicinal chemistry letters 18, 4872–4875 (2008). 18691886 10.1016/j.bmcl.2008.07.071

