# Supplementary material for: Imbalance in chemical space: How to facilitate the identification of protein-protein interaction inhibitors
Source: Sci Rep. 2016 Apr 1;6:23815. doi: 10.1038/srep23815 (PMC4817116; doi:10.1038/srep23815)
Supplement: Supplementary Information [file srep23815-s1.pdf]

Imbalance in chemical space: How to facilitate the identification of protein-protein interactions inhibitors?

Mélaine A. Kuenemann[1,2], Céline M. Labbé[1,2], Adrien H. Cerdan[1,2] , and Olivier Sperandio[1,2,*]
[1] Université Paris Diderot, Sorbonne Paris Cité, Molécules Thérapeutiques In Silico, INSERM UMR-S 973, Paris, France
[2] INSERM, U973, Paris, France
[*] corresponding author

SUPPLEMENTARY MATERIAL

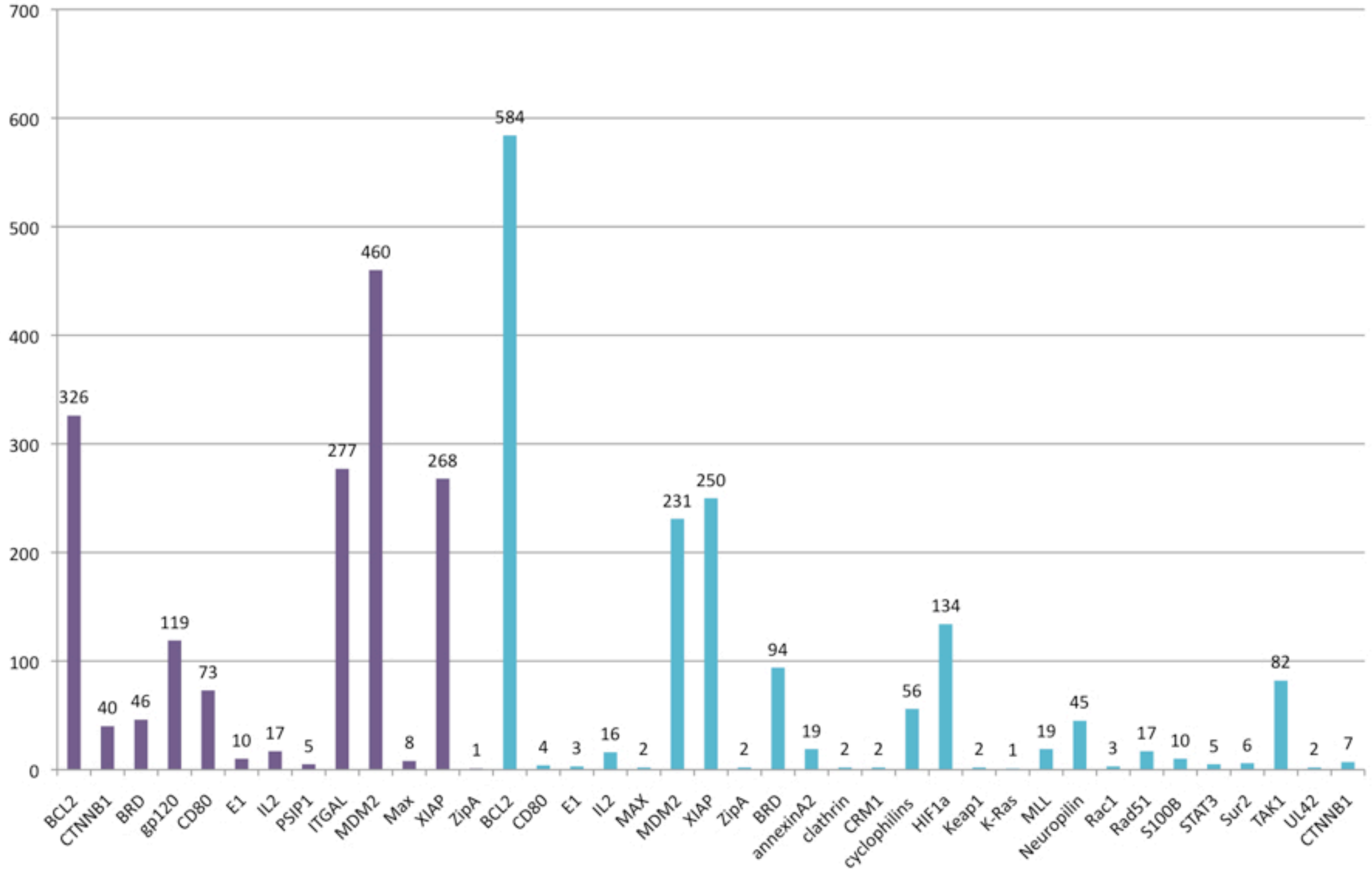
700
600
500
400
300
200
100
0
326
40
46
119
73
10
17
5
277
460
8
268
1
584
4
3
16
2
231
250
2
94
19
2
2
56
134
2
1
19
45
3
17
10
5
6
82
2
7
BCL2
CTNNB1
BRD
gp120
CD80
E1
IL2
PSIP1
ITGAL
MDM2
Max
XIAP
ZipA
BCL2
CD80
E1
IL2
MAX
MDM2
XIAP
ZipA
BRD
annexinA2
clathrin
CRM1
cyclophilins
HIF1a
Keap1
K-Ras
MLL
Neuropilin
Rac1
Rad51
S100B
STAT3
Sur2
TAK1
UL42
CTNNB1

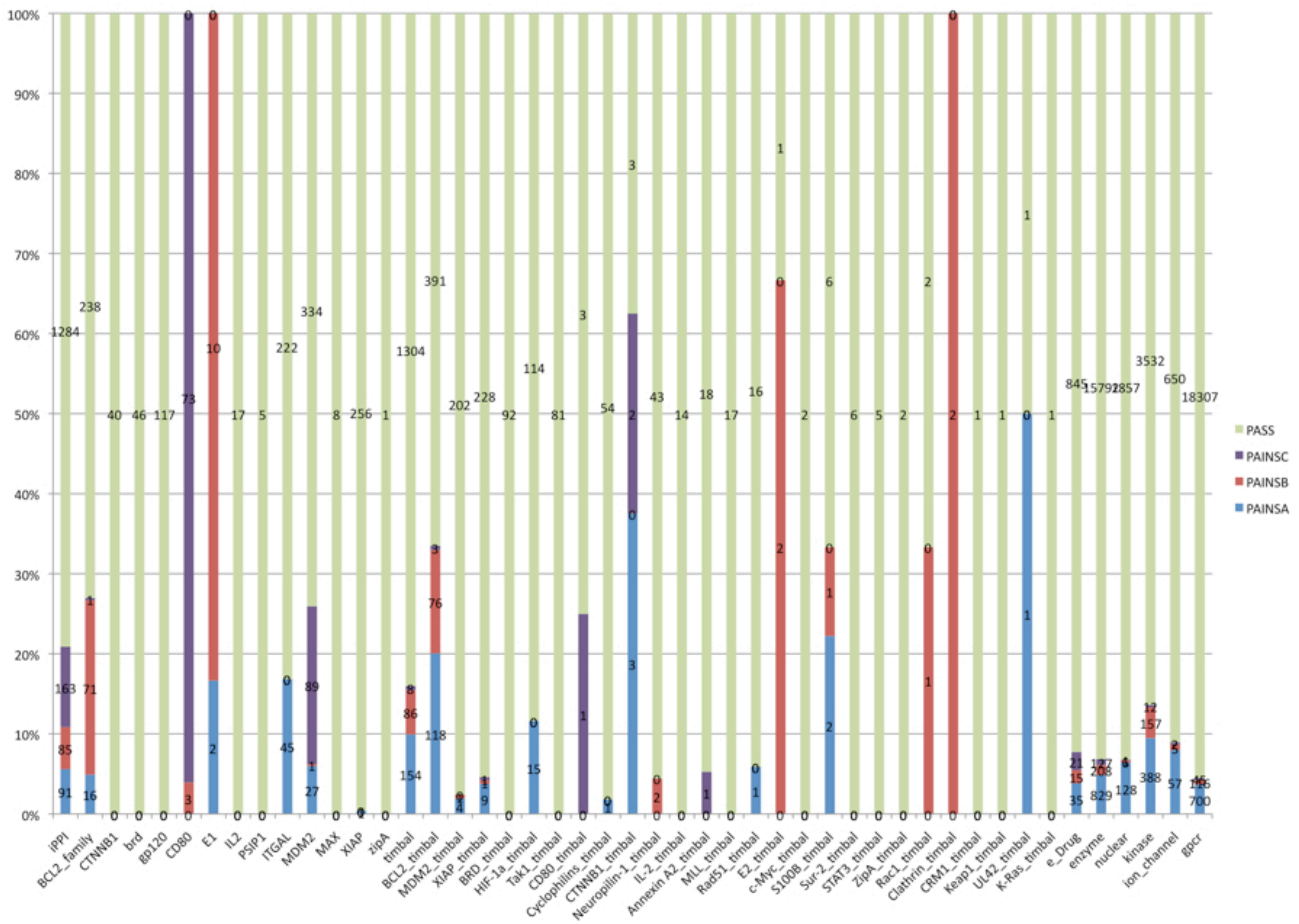
100%
90%
80%
70%
60%
50%
40%
30%
20%
10%
0%
PASS
PAINSC
PAINSB
PAINSA
iPPI
BCL2_family
CTNNB1
brd
gp120
CD80
E1
IL2
PSIP1
ITGAL
MDM2
MAX
XIAP
zipA
timbal
BCL2_timbal
MDM2_timbal
XIAP_timbal
BRD_timbal
HIF-1a_timbal
Tak1_timbal
CD80_timbal
Cyclophilins_timbal
CTNNB1_timbal
Neuropilin-1_timbal
IL-2_timbal
Annexin A2_timbal
MLL_timbal
Rad51_timbal
E2_timbal
c-Myc_timbal
S100B_timbal
Sur-2_timbal
STAT3_timbal
ZipA_timbal
Rac1_timbal
Clathrin_timbal
CRM1_timbal
Keap1_timbal
UL42_timbal
K-Ras_timbal
e_Drug
enzyme
nuclear
kinase
ion_channel
gpcr

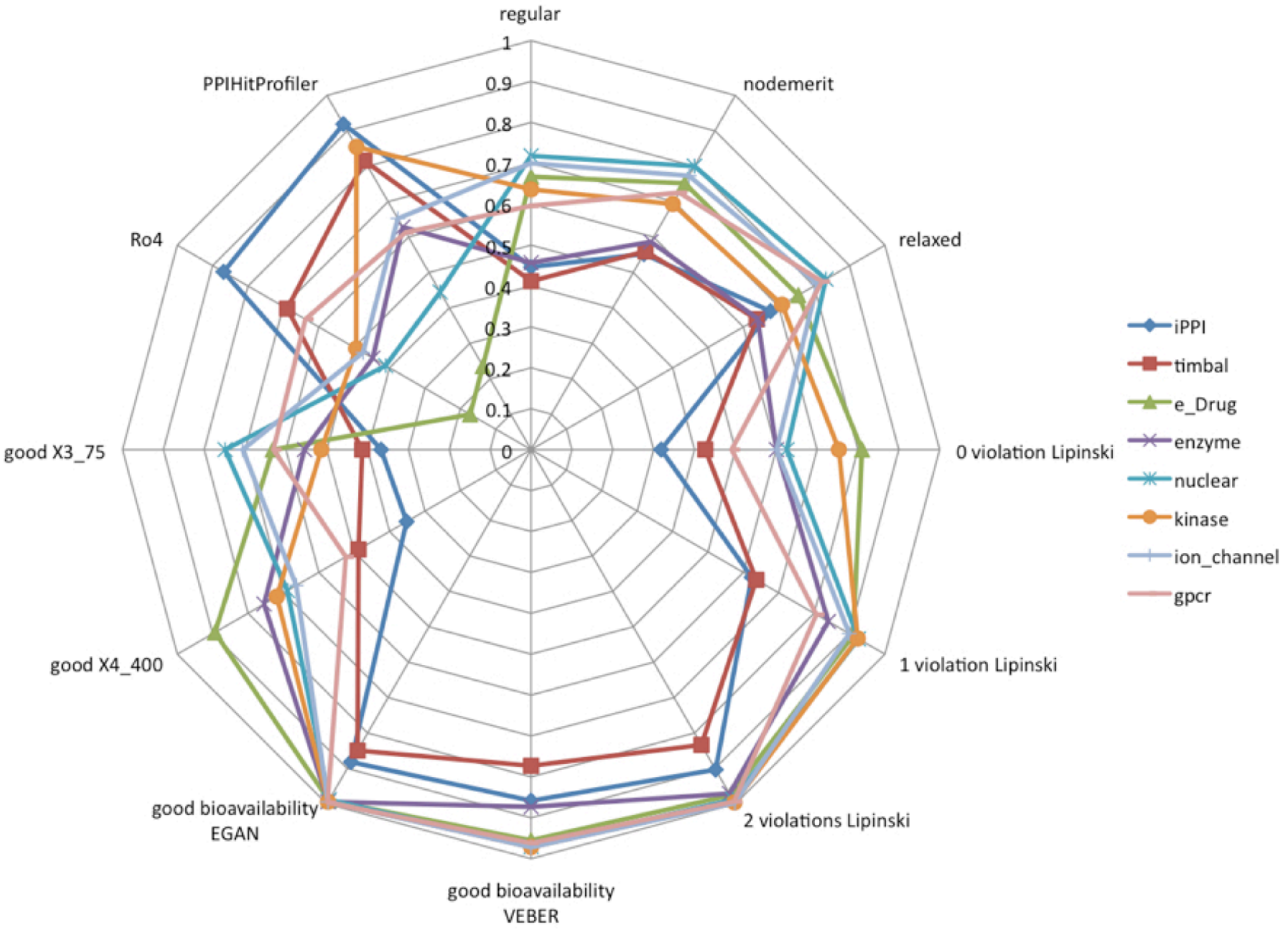
regular
nodemerit
relaxed
0 violation Lipinski
1 violation Lipinski
2 violations Lipinski
good bioavailability VEBER
good bioavailability EGAN
good X4_400
good X3_75
Ro4
PPIHitProfiler
1
0.9
0.8
0.7
0.6
0.5
0.4
0.3
0.2
0.1
0
iPPI
timbal
e_Drug
enzyme
nuclear
kinase
ion_channel
gpcr

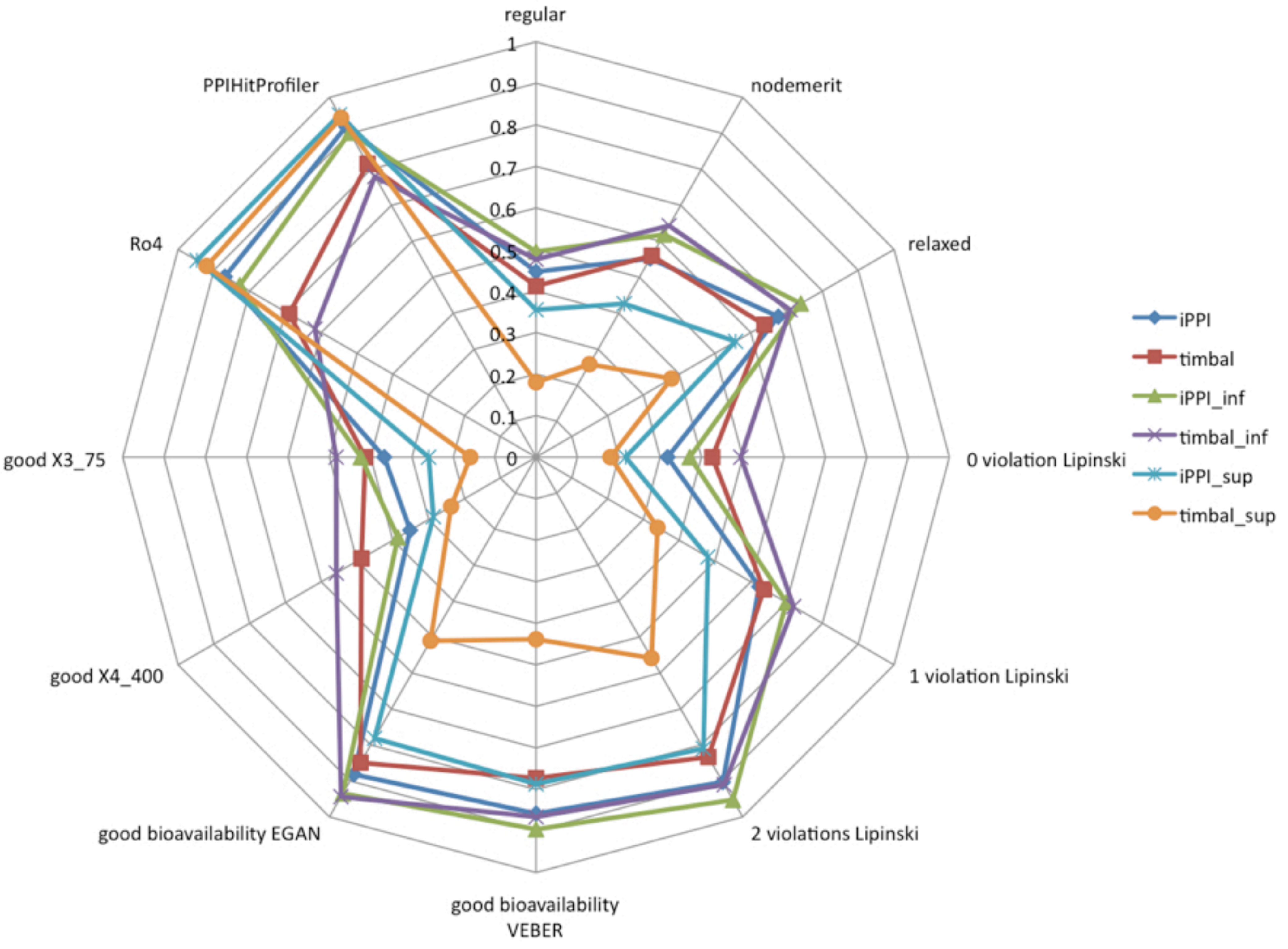
regular
nodemerit
relaxed
0 violation Lipinski
1 violation Lipinski
2 violations Lipinski
good bioavailability VEBER
good bioavailability EGAN
good X4_400
good X3_75
Ro4
PPIHitProfiler
1
0.9
0.8
0.7
0.6
0.5
0.4
0.3
0.2
0.1
0
iPPI
timbal
iPPI_inf
timbal_inf
iPPI_sup
timbal_sup

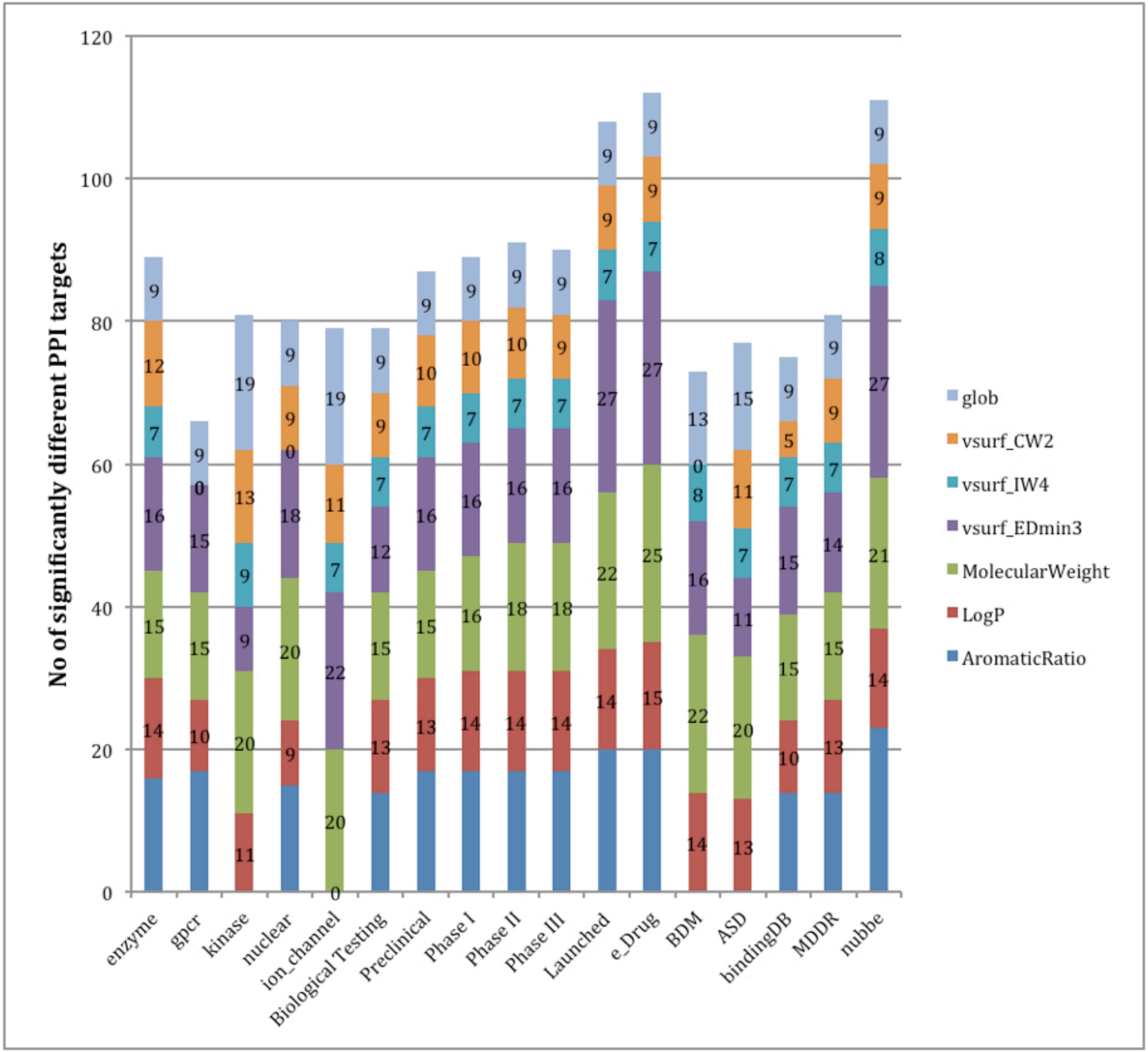
No of significantly different PPI targets
0
20
40
60
80
100
120
enzyme
gpcr
kinase
nuclear
ion_channel
Biological Testing
Preclinical
Phase I
Phase II
Phase III
Launched
e_Drug
BDM
ASD
bindingDB
MDDR
nubbe
glob
vsurf_CW2
vsurf_IW4
vsurf_EDmin3
MolecularWeight
LogP
AromaticRatio

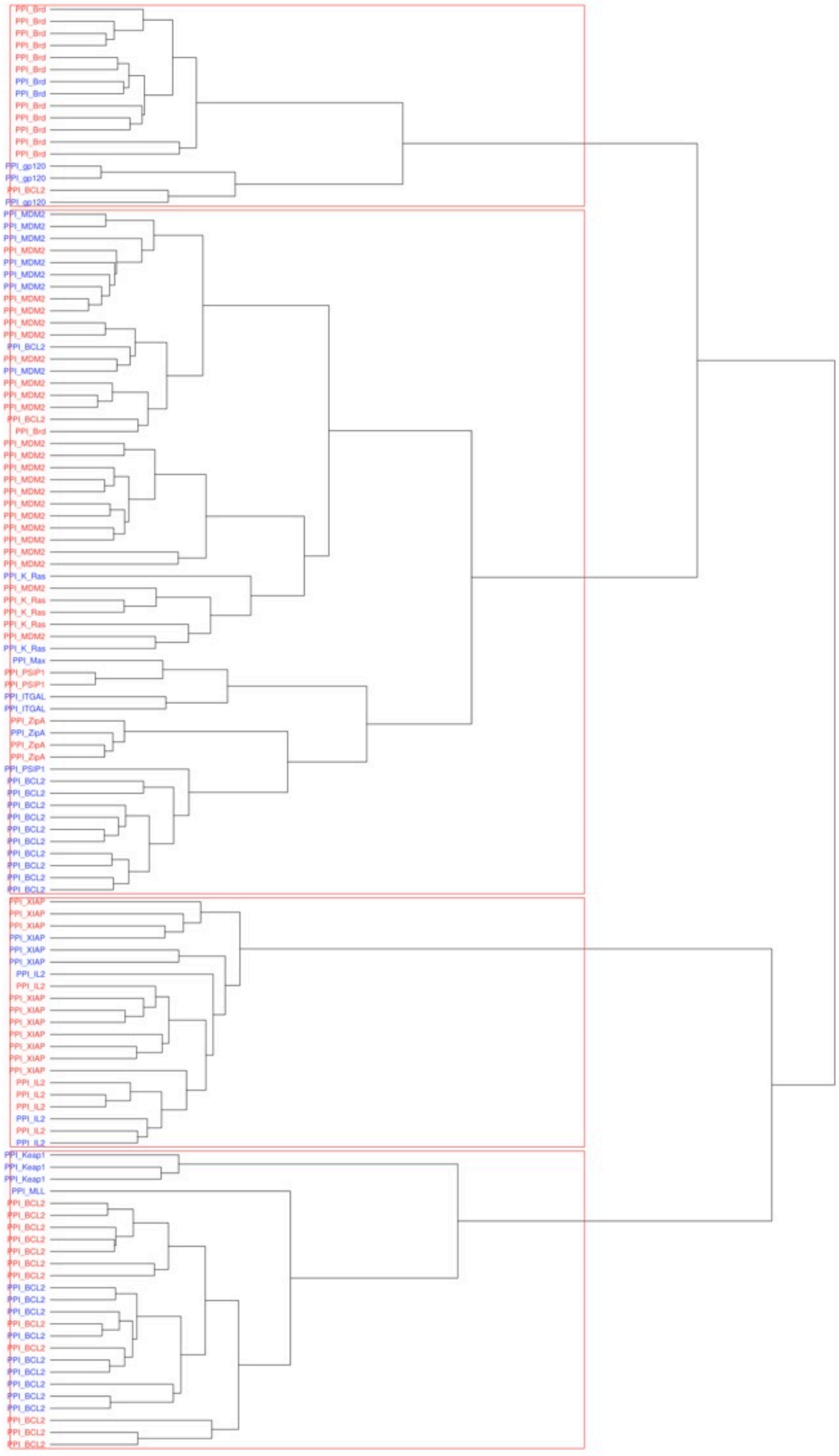
PPI_Brd
PPI_Brd
PPI_Brd
PPI_Brd
PPI_Brd
PPI_Brd
PPI_Brd
PPI_Brd
PPI_Brd
PPI_Brd
PPI_Brd
PPI_Brd
PPI_Brd
PPI_gp120
PPI_gp120
PPI_BCL2
PPI_gp120
PPI_MDM2
PPI_MDM2
PPI_MDM2
PPI_MDM2
PPI_MDM2
PPI_MDM2
PPI_MDM2
PPI_MDM2
PPI_MDM2
PPI_MDM2
PPI_MDM2
PPI_BCL2
PPI_MDM2
PPI_MDM2
PPI_MDM2
PPI_MDM2
PPI_MDM2
PPI_BCL2
PPI_Brd
PPI_MDM2
PPI_MDM2
PPI_MDM2
PPI_MDM2
PPI_MDM2
PPI_MDM2
PPI_MDM2
PPI_MDM2
PPI_MDM2
PPI_MDM2
PPI_MDM2
PPI_K_Ras
PPI_MDM2
PPI_K_Ras
PPI_K_Ras
PPI_K_Ras
PPI_MDM2
PPI_K_Ras
PPI_Max
PPI_PSIP1
PPI_PSIP1
PPI_ITGAL
PPI_ITGAL
PPI_ZipA
PPI_ZipA
PPI_ZipA
PPI_ZipA
PPI_PSIP1
PPI_BCL2
PPI_BCL2
PPI_BCL2
PPI_BCL2
PPI_BCL2
PPI_BCL2
PPI_BCL2
PPI_BCL2
PPI_BCL2
PPI_BCL2
PPI_XIAP
PPI_XIAP
PPI_XIAP
PPI_XIAP
PPI_XIAP
PPI_XIAP
PPI_IL2
PPI_IL2
PPI_XIAP
PPI_XIAP
PPI_XIAP
PPI_XIAP
PPI_XIAP
PPI_XIAP
PPI_XIAP
PPI_IL2
PPI_IL2
PPI_IL2
PPI_IL2
PPI_IL2
PPI_IL2
PPI_Keap1
PPI_Keap1
PPI_Keap1
PPI_MLL
PPI_BCL2
PPI_BCL2
PPI_BCL2
PPI_BCL2
PPI_BCL2
PPI_BCL2
PPI_BCL2
PPI_BCL2
PPI_BCL2
PPI_BCL2
PPI_BCL2
PPI_BCL2
PPI_BCL2
PPI_BCL2
PPI_BCL2
PPI_BCL2
PPI_BCL2
PPI_BCL2
PPI_BCL2
PPI_BCL2
PPI_BCL2

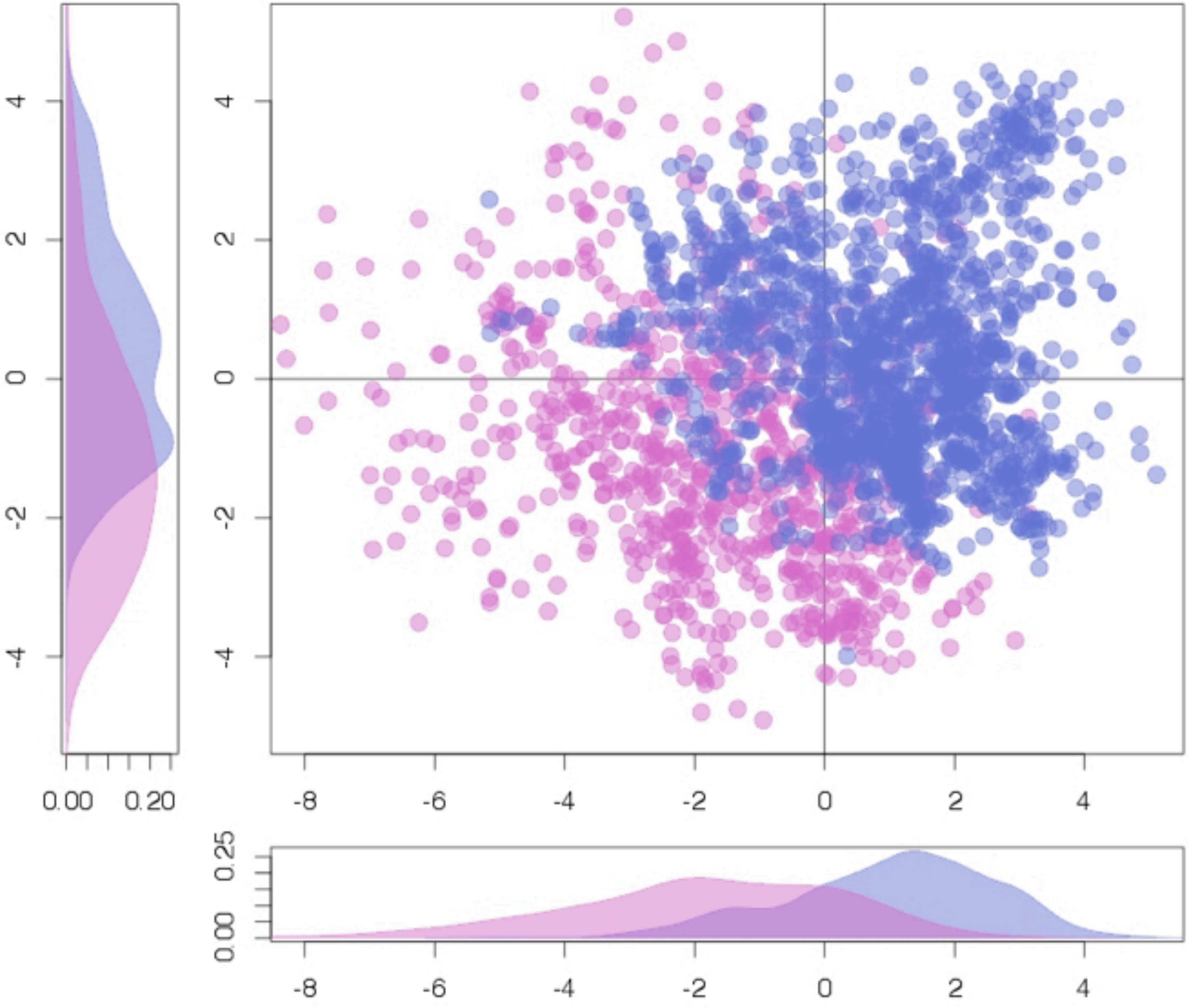
4
2
0
-2
-4
0.00
0.20
-8
-6
-4
-2
0
2
4
0.25
0.00

Figure S1: Distribution of iPPI compounds across the PPI target space for both iPPI-DB (violet) and TIMBAL (blue).

Figure S2: Distribution of the PAINS substructures that were detected in the iPPI compound dataset using the FAF-Drugs3 web server. Four bins are defined per PPI target, which contains the number of compounds that have either the PASS or PAINS filter or are flagged by one of the 3 filters: PAINS-A, PAINS-B, or PAINS-C, corresponding to the number of occurrences that a given substructure has been present in an active compound within a bioassay with the following correspondence: PAINS-A>150 times; 15 times<PAINS-B<150 times; and PAINS-C<15 times.

Figure S3: Radar chart displaying the level of compliance of the iPPI datasets and some key reference compound datasets vis à vis some chemistry rules that are commonly used to assess the pharmacokinetic profiles of drug candidates and the compound propensity for being a putative iPPI (Ro4 and PPI-HitProfiler).

Figure S4: Radar chart displaying the level of compliance of the iPPI datasets when subdivided into two bins of activity, Inf: $pXC_{50}<7$, and Sup: $pXC_{50}>7$.

Figure S5: Number of significantly different PPI targets for all of the descriptors.

Figure S6: Dendrogram resulting from the pocket-driven classification of all of the available PPI crystal structures using a set of 117 pocket descriptors.

Figure S7: Example of a PCA between two populations: iPPI-DB (in blue) and e_Drugs (in pink);
the densities on each component are indicated.
